# Tailored Stereo‐Configuration of Crosslinkable Polyester‐Based Solid Polymer Electrolytes for All‐Solid‐State and Binder‐Free Li Metal Batteries

**DOI:** 10.1002/advs.202600063

**Published:** 2026-08-24

**Authors:** Hyun Jun Song, Jeong Yoon Kim, Sungpyo Hong, Yong Hui Kim, Myeong Gyun Nam, Jeong‐Won Ho, Min Gi Kang, Hye Seon Kim, Chaeyeon Ha, Sang Uck Lee, Pil J. Yoo, Patrice Rannou

**Affiliations:** ^1^ School of Chemical Engineering Sungkyunkwan University (SKKU) Suwon Republic of Korea; ^2^ SKKU Advanced Institute of Nanotechnology (SAINT) Sungkyunkwan University (SKKU) Suwon Republic of Korea; ^3^ SKKU National Lab for Intelligent Energy Solution Technology (SIEST) Sungkyunkwan University (SKKU) Suwon Republic of Korea; ^4^ Université Grenoble Alpes Université Savoie Mont‐Blanc CNRS Grenoble‐INP LEPMI Grenoble France

**Keywords:** end‐group fluorination, photo‐crosslinking, solid polymer electrolytes, solvation structure regulation, stereoisomerism

## Abstract

By harnessing stereoisomerism in unsaturated polyesters, we elucidate how subtle structural variations impact Li^+^ solvation structures by modulating free volume, Li^+^ diffusivity, and lithium salt dissociation within solid polymer electrolytes (SPEs). The intrinsic reactivity of C═C bonds along the polymeric backbones is further exploited via a facile UV‐curing approach to construct robust three‐dimensional polymeric networks that impart mechanical robustness and preferential restriction of TFSI^−^ mobility relative to Li^+^ transport. The distorted conformation of poly(*n*‐pentyl maleate) (PPM) leads to sparsely crosslinked structures, preserving efficient Li^+^ transport pathways. Furthermore, end‐group fluorination enhances interfacial stability by facilitating the formation of a LiF‐rich solid electrolyte interphase. These synergistic design strategies culminate in a crosslinked PPM‐based SPE (XL‐PPM‐3F‐Li) featuring one CF_3_ end‐capping group, which exhibits remarkable electrochemical durability in both Li||Li symmetric and LFP full‐cell configurations. Furthermore, this advantage is extended to the device level by integrating binder, electrolyte, and separator into a single ultrathin (∼“5 µm‐thick”) crosslinked SPE that conformally interfaces with the electrode microstructure. This integrated configuration markedly lowers interfacial and charge‐transfer resistance, enabling 65% of the initial reversible capacity at 2 C and 93.6% retention after 100 cycles at 0.5 C (60°C). Overall, this crosslinking‐enabled unified architecture establishes a robust strategy for ultra‐stable solid‐state lithium‐metal batteries.

## Graphical abstract

Variations in polymer conformations of unsaturated poly(*n*‐alkyl esters) driven by monomer‐level stereochemistry directly govern Li^+^ solvation structures in polymer host matrices. The synergistic strategies of polymer conformation control, UV‐induced interchain crosslinking, and end‐group fluorination are implemented for engineering advanced solid polymer electrolytes for lithium metal batteries.

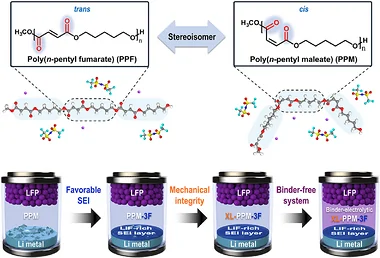

## 1 Introduction

With increasing demand for *safer‐by‐design*, high‐energy‐density post‐Lithium‐ion batteries (LiBs) in electric transportation and large‐scale energy storage systems, extensive effort has been devoted to overcome the intrinsic limitations of conventional LiB systems relying on flammable liquid electrolytes, which pose severe safety concerns and constraints in achievable energy density targets [1, 2, 3, 4, 5, 6, 7]. Solid‐state electrolytes (SSEs) have emerged as promising alternatives, offering enhanced safety and higher energy densities by enabling high‐capacity electrodes. Among SSEs, solid polymer electrolytes (SPEs) are highly attractive for next‐generation all‐solid‐state batteries (ASSBs) due to their superior mechanical flexibility, interfacial compatibility with electrodes, and scalability for large‐scale production [5, 8, 9].

Previous research efforts in SPEs have mainly focused on polyether‐based host matrices for lithium salts, represented by poly(ethylene oxide) (PEO) [10, 11, 12, 13]. However, they suffer from severe drawbacks: low room‐temperature ionic conductivity (*σ*) in the range of 10^−8–^10^−6^ S cm^−1^ due to their high crystallinity, a low Li^+^ cationic transference number ($t_{Li\;}^{+}$≤ 0.2), and a narrow electrochemical stability window (ESW, <4.0 V vs. Li°/Li^+^) [14, 15, 16, 17, 18, 19]. To go beyond PEO‐based SPEs, recent research endeavors identified carbonyl‐coordinating polymer host matrices forming multichain Li^+^ solvation structures, which not only facilitate Li^+^ dissociation by moderate coordination of Li^+^ cations with carbonyl oxygens but also exhibit high oxidative stability. Following reports on poly(*n*‐pentyl malonate), aliphatic polyester‐based SPEs have been systematically explored. X‐ray scattering, molecular dynamics (MD) simulations, and electrochemical characterizations resolved carbonyl‐driven solvation cages and weaker cation–anion correlations [20, 21, 22, 23]. Concurrently, tuning monomers’ chemical structures to introduce molecular asymmetry and deploying fluorine‐rich passivating end‐groups modulate chain packing and stabilize the solid electrolyte interface (SEI) [24, 25].

Nonetheless, post‐PEO SPEs still face challenges with Li° dendrite formation coupled with insufficient mechanical strength. These limitations can be mitigated through crosslinking strategies that generate robust three‐dimensional polymer networks, thereby increasing the Young modulus and resisting dendrite penetration [26, 27]. In addition, covalently crosslinked polymer networks effectively restrict the diffusion of bulky anions of lithium salts, which boosts $t_{Li}^{+}$ and thereby alleviates the rise in interfacial resistance arising from concentration polarization during battery operation [28, 29]. Representative approaches to fabricate crosslinked SPEs (XL‐SPEs) include radical polymerization of acrylates, ring‐opening of epoxy groups, and thiol‐ene click chemistry with multi‐functional thiols, where the reactive groups in telechelic, ionophilic polymer/oligomer bridges or multifunctional nodes are exploited to form interconnected structures triggered by UV irradiation or heating [30, 31]. Extending these research directions, exploring unsaturated polymer host matrices with inherent crosslinking capability, combined with effective crosslinking strategies, presents a versatile pathway toward further advanced SPEs.

Here, we regulated the Li^+^ solvatio n structure of two unsaturated aliphatic polyester derivative host matrices, poly(*n*‐pentyl fumarate) (PPF) and poly(*n*‐pentyl maleate) (PPM), through three synergistic approaches: (i) monomer‐level stereochemistry, (ii) interchain crosslinking, and (iii) end‐group modification. The stereoisomeric variation between PPF (*trans*) and PPM *(cis*) resulted in markedly distinct polymer conformations and Li^+^ solvation structures. The C═C bonds within the repeating unit of PPF and PPM enable photo‐crosslinking to enhance mechanical integrity and the $t_{Li}^{+}$ by selectively hindering the transport of bulky TFSI^−^ anions. Furthermore, trifluoroacetate (–CF_3_) end‐groups were introduced to facilitate more favorable Li^+^ solvation environments and the formation of a stable, LiF‐enriched SEI onto the Li° surface. Consequently, the optimized PPM‐based crosslinked SPE (XL‐PPM‐3F‐Li) achieved a Li^+^ conductivity ($\sigma_{Li^{+}}=\sigma \times \;t_{Li\;}^{+}$) of ∼0.1 mS cm^−1^ at 80°C, with excellent cycling stability in an LFP||XL‐PPM‐3F‐Li||Li° full‐cell configuration.

Beyond its use as a simple electrolyte, we further demonstrate, for the first time, a binder–electrolyte–separator unified system in which the XL‐SPE singularly fulfills simultaneously the roles of binder and separator. The introduction of an ultrathin XL‐SPE film that conforms closely to the electrode microstructure substantially reduces electrode resistance arising from insulating binder domains and also lowers both ionic and charge transfer resistance by improving the electrolyte and electrode interfacial contact. As a result, this integrated system demonstrated markedly improved rate capability, delivering 65% of initial capacity even at a high rate of 2 C, and achieved a capacity retention of 93.6% after 100 cycles in a lithium metal battery. This unification approach integrating binder, electrolytes, and separator through regulated photo‐crosslinking of SPEs holds potential as a promising design platform for next‐generation high‐performance all‐solid‐state lithium‐metal batteries (ASSLMB).

## 2 Results and Discussion

### 2.1 Design Strategies for Crosslinkable Polyester‐Based SPEs

A transesterification A_2_B_2_ polycondensation route was employed for facile upscaling of a series of selected dimethyl esters with 1,5‐pentanediol, yielding aliphatic polyester‐based host matrices for lithium salts, as shown in Figure 1a. Starting from the saturated model, poly(*n*‐pentyl succinate) (PPS), unsaturated C═C moieties were subsequently introduced into the polymer repeating units, producing poly(*n*‐pentyl fumarate) (PPF) and poly(*n*‐pentyl maleate) (PPM), which allow interchain crosslinking via UV‐induced [2+2] cycloaddition reaction. Notably, PPF and PPM were derived from stereoisomeric monomers, dimethyl fumarate (*trans*) and dimethyl maleate (*cis*), respectively. Additionally, the reactive hydroxyl (−OH) end‐groups seated at one end of their hemitelechelic macromolecular architectures were converted to –CF_3_ functions (denoted as ‐3F) using trifluoroacetic anhydride (TFAA). ^19^F NMR spectroscopic analysis verified that the conversion rate of the terminal −OH groups exceeded 70% (Figures S1 and S2).

**FIGURE 1:**
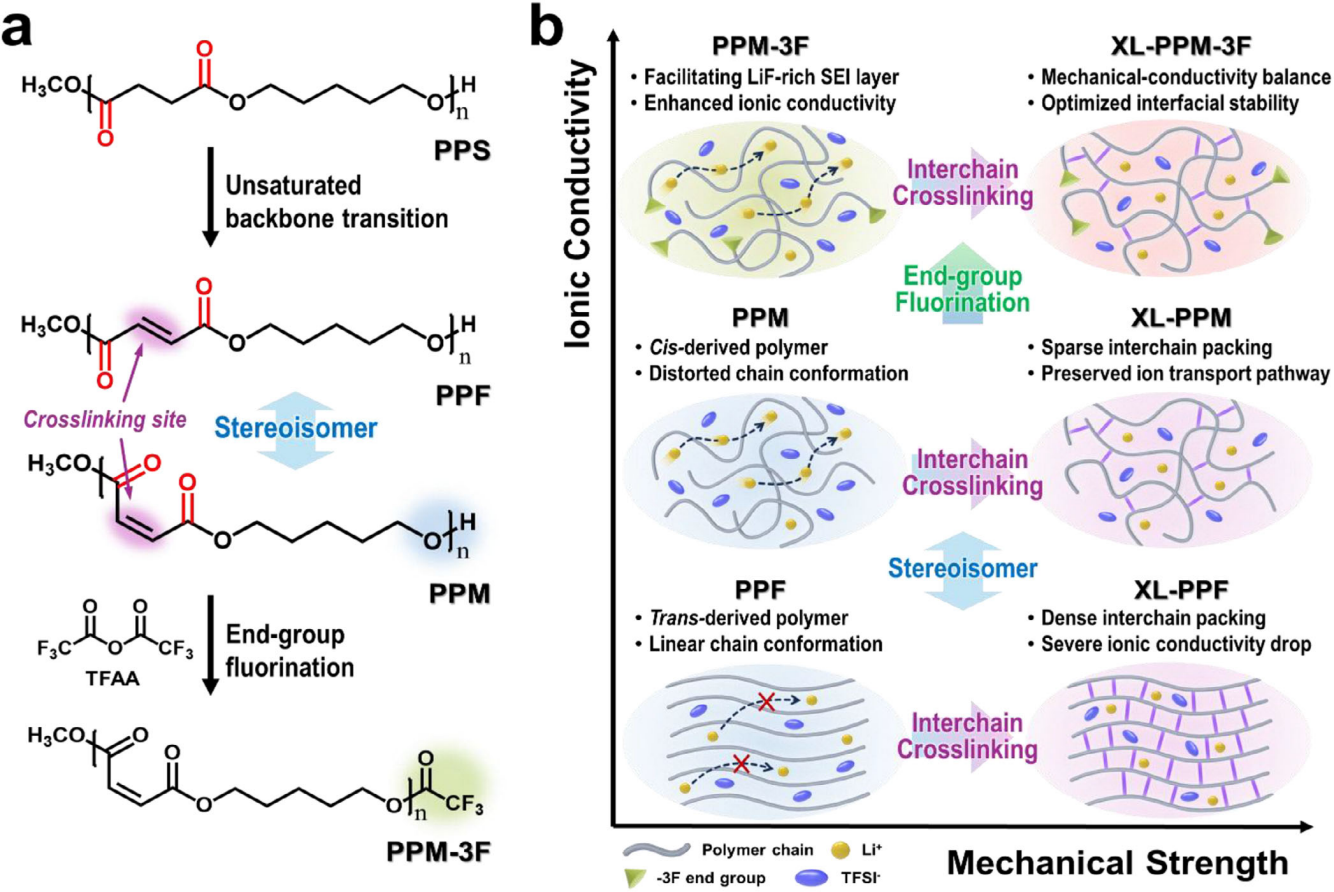
(a) Chemical structures of the synthesized polyester‐based host matrices. (b) Schematics showing effects of the stereoisomerism, end‐group fluorination, and interchain crosslinking in the polyester‐based host matrices with respect to ionic conductivity and mechanical strength properties.

Figure 1b presents a schematic summary of how the structural differences among the synthesized polymer host matrices influence their electrolyte performance. The geometric difference between PPF and PPM directly affects their chain conformations, leading to distinct variations in the Li^+^ solvation structures in *salt‐in‐polymer* SPEs. The *trans*‐derived PPF adopts a linear chain conformation, which enables well‐ordered interchain packing. In contrast, the *cis*‐derived PPM exhibits a distorted chain geometry, leading to a disordered packing arrangement. This disordered interchain organization provides more accessible free volume for ion transport compared to PPF, thereby imparting higher ionic conductivity to PPM.

The pronounced conformational differences between these two stereoisomeric polymers also led to clearly distinguished network structures after interchain crosslinking. The *cis*‐conformation of PPM introduces an intrinsic backbone bending and conformational distortions, which significantly reduce interchain alignment and increase the average spatial separation between neighboring polymer chains. As a result, the spatial proximity between crosslinking sites is lowered, leading to a reduced crosslinking probability under identical reaction conditions [32]. Consequently, although the same crosslinking chemistry is applied to both polymers, the distorted chain geometry of PPM intrinsically limits efficient interchain junction formation, giving rise to a loosely‐connected and sparsely‐crosslinked network in which ion transport pathways remain largely intact even after crosslinking. In contrast, the linear *trans*‐derived PPF chains allow for closer interchain packing and more frequent crosslinking events, resulting in a denser polymeric network that restricts ion transport pathways and results in a substantial reduction in ionic conductivity. This structural characteristic allows PPM to mitigate the typical tradeoff between ionic conductivity and mechanical strength that is commonly observed in conventional crosslinked SPEs. In addition, fluorination of the hemitelechelic polymer end‐groups improves interfacial stability by forming a LiF‐enriched SEI on the lithium metal anode and promotes a favorable Li^+^ solvation environment by enhancing LiTFSI dissociation. As a result, XL‐PPM‐3F exhibits a well‐balanced set of properties, including high Li^+^ conductivity, sufficient mechanical robustness to suppress Li° dendrite growth, and the ability to form a stable interfacial layer at the electrode surface, which eventually demonstrates its strong potential as an optimized SPE.

### 2.2 Thermal and Physicochemical Characterization

As shown in Figure 2a and Figure S3, thermogravimetric analyses (TGA) were conducted to determine the thermal decomposition temperatures (*T_dec_*) of the polyesters and their *salt‐in‐polymer* electrolytes containing 25 wt.% LiTFSI (the corresponding [O]/[Li] molar ratio (*r*) is 18, as summarized in Table S1), hereinafter denoted as –Li. To ensure minimal influence from molar mass effects on their physicochemical and thermal properties, the number‐average degree of polymerization (DP_n_) of all synthesized polyesters was verified to be comparable (ca. 33–35 repeating units) through nuclear magnetic resonance (NMR) and size‐exclusion chromatography (SEC) analyses (Table S2 and Figures S4 and S6). For pristine polymers (without LiTFSI), a slight increase in *T_dec_* after crosslinking polymer matrices indicates that the covalently interconnected network structures enhance their thermal stability by delaying chemical degradation of polymer chains. In contrast, the incorporation of LiTFSI into the polymer matrices resulted in a reduction in *T_dec_*, which is likely due to the coordination of Li^+^ cations with carbonyl oxygens, enhancing the chemical reactivity of the polymers.

**FIGURE 2:**
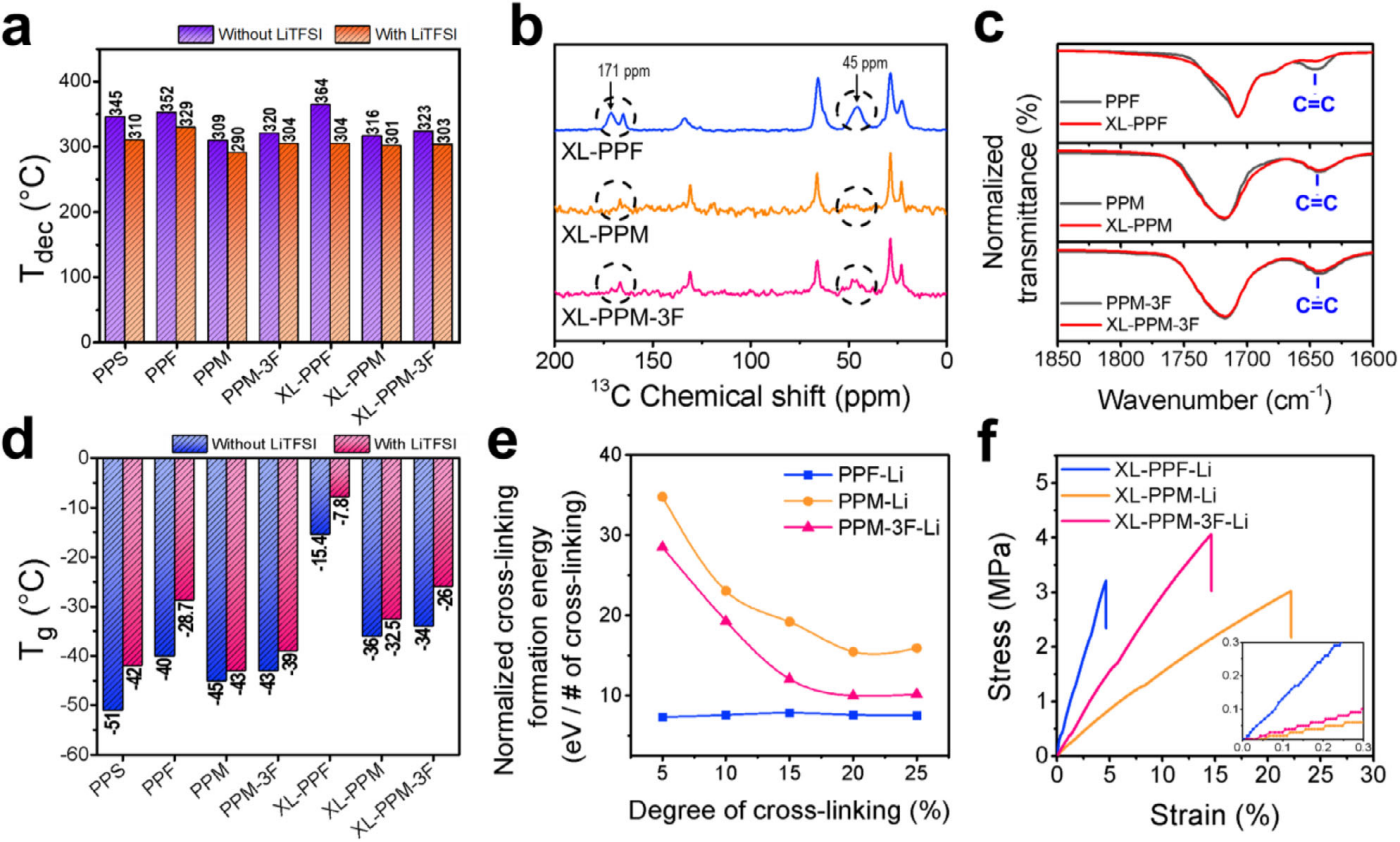
(a) *T_dec_* of the synthesized poly(*n*‐alkyl esters) with and without LiTFSI, extracted from TGA analyses. (b) Solid‐state ^13^C NMR spectra of crosslinked polymer thin films. (c) FTIR spectra of the polymer host matrices, before and after crosslinking. (d) *T_g_* of the poly(*n*‐alkyl esters) with and without LiTFSI, extracted from DSC analyses. (e) Normalized crosslinking formation energy of the SPEs calculated from MD simulations. (f) Stress–strain curves of XL‐SPEs obtained from tensile testing.

Differential scanning calorimetry (DSC) analyses (Figure 2d and Figure S8) elucidated the key thermal properties of the polyesters and their *salt‐in‐polymer* electrolytes with the same LiTFSI contents as the samples used in TGA measurements. In case of PPS, the pristine polymer shows a glass transition temperature (*T_g_*) at ca. −51°C and a melting point (*T_m_*) of ca. 34°C. When LiTFSI is incorporated into PPS, the lithium salt plasticizes the PPS, resulting in the disappearance of the *T_m_* and the appearance of only the *T_g_*, indicating an amorphous nature. Both PPM and PPM‐Li were found to be fully amorphous, each displaying only *T_g_* without *T_m_*. In contrast, PPF and PPF‐Li exhibited semi‐crystalline features, with distinct *T_m_* at ca. 66°C (Figure S9). Upon crosslinking of PPM and PPF, neat polymers and their SPE analogs exhibited amorphous nature with increased *T_g_* compared to their uncrosslinked counterparts, consistent with restricted segmental mobility. Notably, the *T_g_* of PPF increased by ca. 20°C, approximately twice the *ΔT_g_* observed for the PPM derivatives, reflecting the higher crosslinking density of the PPF polymer network, which originates from its more linear chain conformation.

Solid‐state ^13^C NMR and Fourier‐transform infrared (FTIR) spectroscopic characterizations were conducted to investigate structural changes upon photo‐crosslinking. In Figure 2b, compared with PPM, the spectrum of crosslinked PPF exhibited a new resonance at ca. 45 ppm, which can be attributed to the formation of interchain C─C bonds generated during the chemical crosslinking process. In addition, unlike PPM, PPF displayed an additional peak at ca. 171 ppm. By referring to the ^13^C chemical shift assignments of PPS (Figure S4a), this resonance can be attributed to a C═O function. Specifically, it corresponds to the C═O adjacent to a single bond, rather than the one located next to the original C═C double bond (ca. 165 ppm). Since the double bonds in PPF participated in the crosslinking reaction, the resonance at 171 ppm is assigned to carbonyl groups that remain intact but experienced a distinct chemical environment upon crosslinking [33]. FTIR spectra in Figure 2c also confirmed the distinct crosslinking degrees between PPM and PPF (Figure 2b). Upon photo‐crosslinking, the intensity of the C═C stretching vibration band (ca. 1645 cm^−^^1^) was markedly reduced in PPF, indicative of a high crosslinking efficiency [34]. By contrast, PPM and PPM‐3F exhibited only marginal decreases in this IR band, suggesting the formation of comparatively sparse crosslinked networks. MD simulations supported these findings, revealing a lower crosslinking formation energy for PPF‐Li (Figure 2e). This allows forming a densely‐crosslinked brittle thin film, whereas the higher energy for PPM‐Li results instead in a flexible thin film (Figure S10). Accordingly, crosslinking content in Table S3 suggests that XL‐PPF has the highest crosslinking efficiency, as inferred from its crosslinking content of 97.9%, followed by XL‐PPM‐3F (60.1%) and XL‐PPM (57.5%). The contrasting crosslinking behavior was reflected in their mechanical properties, as evidenced by tensile testing (Figure 2f). XL‐PPF‐Li displayed a Young modulus of ca. 123 MPa, while XL‐PPM‐Li exhibited a six times lower value with improved elongation limit (Table S4).

### 2.3 Computational Simulations and Electrochemical Characterizations

To elucidate the correlation between polymer structure and ionic transport characteristics, density functional theory (DFT) and MD simulations were performed. The composition of each simulated system was defined based on the DP_n_ values of the polyesters determined from SEC and NMR analyses, with the optimized *r* values fixed at 18 for all SPE systems (Figure S11).

As shown in Figure 3a, PPM and PPM‐3F exhibited significantly distorted backbone conformations, unlike the relatively linear backbones of PPS and PPF. This structural distortion originated from the *cis*‐configuration of polymeric repeating units of PPM yield disordered packing of polymer chains, thereby disrupting interchain aggregation and increasing the fractional free volume within the polymeric host matrices, as quantitatively confirmed by MD simulations (Figure 3b). As described in Figure 3c, the calculated free volume fractions of PPM‐ and PPM‐3F‐based systems were substantially higher than those of PPS‐ and PPF‐based counterparts, providing more accessible channels for ion migration. Consistently, the diffusion coefficients extracted from the mean‐square displacement (MSD) profiles (Figure S12 and Table S5) suggest more rapid Li^+^ cation migration within PPM and PPM‐3F host matrices than in PPS or PPF.

**FIGURE 3:**
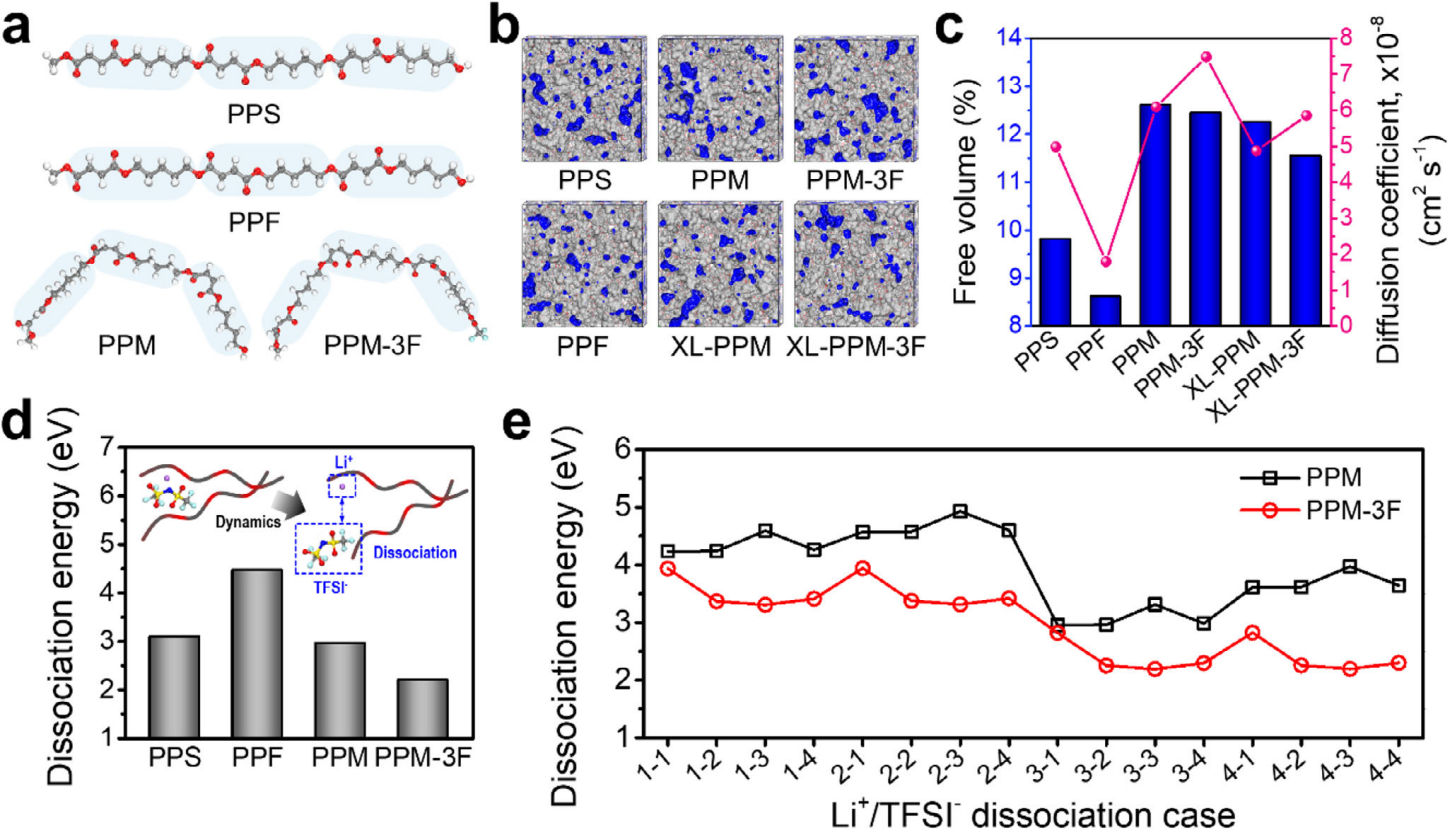
(a) Chain fragments of the polymer host matrices relaxed using DFT calculations. (b) Visual representation of free volume distributions in the SPEs. (c) Free volume and Li^+^ diffusion coefficient in the SPEs at 80°C calculated by MD simulations. (d) Li^+^–TFSI^−^ dissociation energies within the different polymer host matrices obtained from DFT simulations and a schematic illustration of the Li^+^–TFSI^−^ dissociation process. (e) Li^+^–TFSI^−^ dissociation energy at each possible coordination site within the PPM and PPM‐3F host matrices simulated by DFT.

To further clarify the influence of polymer‐salt interactions on ionic transport, the LiTFSI dissociation energies were quantitatively evaluated for all the polymeric host matrices (Figure 3d) according to:

(1) $$\begin{array}{ccc} E_{Dissociation\;energy} & = & E_{Li^{+}+polymer}+E_{TFSI^{-}+polymer}\\ & & -\;\left(E_{polymer}+E_{Li^{+}-TFSI^{-}+polymer}\right) \end{array}$$

Among the series of polymer host matrices, PPF exhibited the highest dissociation energies (> 4.4 eV), indicative of strong Li^+^–TFSI^−^ association within their densely packed and less polar backbones. In contrast, PPM and PPM‐3F showed substantially lower dissociation energies, suggesting weaker Coulombic interactions and a higher tendency for ion pair separation. The weakened Li^+^–TFSI^−^ association in these polymer host matrices can be attributed to the presence of multiple carbonyl coordination sites and enhanced segmental mobility derived from their more distorted chains. To further delineate the distinct behavior between PPM and PPM‐3F polymer host matrices, dissociation energies were systematically evaluated by placing Li^+^ and TFSI^−^ at various coordinating positions along the polymer backbones as shown in Figure S13. To assess the sensitivity to charge scaling, MD simulations were performed using different charge‐scaling factors of 0.6, 0.7, and 0.8 while keeping all other simulation and analysis conditions unchanged. Interestingly, although PPM possesses a slightly higher free volume than PPM‐3F, it displays stronger Li^+^–TFSI^−^ binding across all cases, consistent with its higher average dissociation energies (3.2–4.6 eV for PPM vs. 2.2–3.8 eV for PPM‐3F). It is also noted that PPM‐3F exhibited a higher Li^+^ diffusion coefficient than PPM at all three scaling factors, which strongly supports the experimentally observed ionic/Li^+^ conductivity values (σ and $\sigma_{Li^{+}}$) (Figure 4g). The electron‐withdrawing–CF_3_ end groups of PPM‐3F polarize adjacent carbonyl oxygens, weakening Li^+^–TFSI^−^ coordination and facilitating spontaneous ion dissociation. ^19^F NMR spectroscopic analysis revealed that the –CF_3_ resonance undergoes a noticeable chemical shift upon LiTFSI addition, suggesting a modification of the local electronic environment induced by the electron‐withdrawing end‐groups (Figure S2). Consequently, PPM‐3F generates a larger population of free charge carriers, effectively compensating for its smaller free volume with respect to the one of PPM and yielding superior ionic transport characteristics.

**FIGURE 4:**
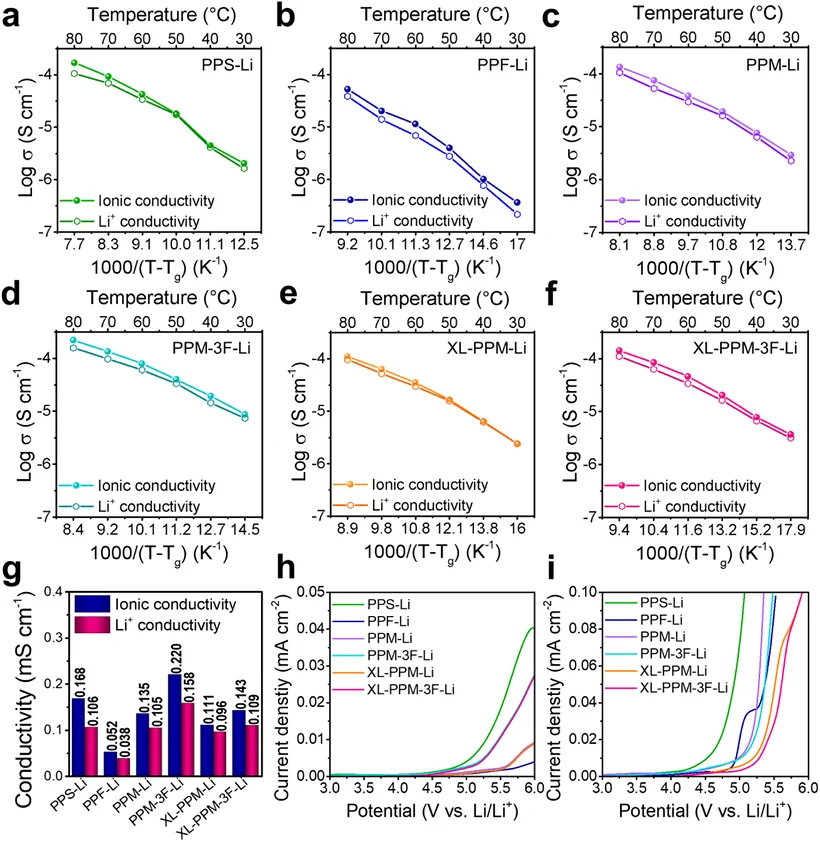
VFT plots of ionic conductivity (*σ*) and Li^+^ conductivity ($\sigma_{Li^{+}}$) of (a) PPS‐Li, (b) PPF‐Li, (c) PPM‐Li, (d) PPM‐3F‐Li, (e) XL‐PPM‐Li, and (f) XL‐PPM‐3F‐Li at 30°C–80°C. (g) Ionic conductivity (*σ*) and Li^+^ conductivity ($\sigma_{Li^{+}}$) of SPEs at 80°C. LSV curves of the SPEs at (h) 30°C and (i) 80°C.

The SPEs displayed temperature‐dependent ionic conductivities that conformed well to the Vogel–Fulcher–Tammann (VFT) model across all LiTFSI contents (Figure 4a–f, and Figures S14 and S15). Maximum *σ* was achieved at 80°C with optimized *r* values of 18 for all SPEs. As shown in Figures S16 and S17, all SPEs exhibited high $t_{Li}^{+}$ exceeding 0.6, i.e. more than six times greater values than the one of mPEG‐Li (Figure S18). In particular, XL‐PPM‐Li and XL‐PPM‐3F‐Li demonstrated exceptional $t_{Li}^{+}$ of 0.87 and 0.74, respectively, at 80°C, which is highly beneficial for mitigating concentration polarization during cycling of SSBs. This was further supported by variable‐gradient pulsed‐field‐gradient (PFG) NMR analysis (Figure S18). After crosslinking of PPM‐3F‐Li, TFSI^−^ diffusivity was reduced more strongly than Li^+^ diffusivity, resulting in an increase in the self‐diffusion‐based Li^+^ fraction, $D_{Li^{+}}/\left(D_{Li^{+}}+D_{\mathrm{TFS}I^{-}}\right)$, from 0.575 for PPM‐3F‐Li to 0.637 for XL‐PPM‐3F‐Li. This trend agrees well with the electrochemically observed increase in $t_{Li}^{+}$, supporting that crosslinking preferentially restricts anion motion and thereby enhances Li‐selective ion transport.

The ESWs against Li°/Li^+^ of the SPEs were evaluated using linear sweep voltammetry (LSV) (Figure 4h,i). All tested SPEs displayed oxidation onset values 1.5–2.3 V higher than the one displayed by PEO‐Li (Figure S19). Intriguingly, PPS‐Li exhibited the lowest oxidation voltage among the SPEs, contrary to the conventional expectation that C═C bonds are more reactive (Figure S20). To further analyze the effect of C═C moieties in polymeric backbones on their oxidative stability, rheological and FTIR analyses were conducted after LSV measurements (Figures S21 and S22). In Figure S21, frequency‐sweep oscillatory rheology (γ = 1%, 0.1–100 Hz, 80°C) was performed on PPS‐Li and PPF‐Li before and after 10 repeated LSV cycles. Electrochemical oxidation process and subsequent rheological analyses were conducted at 80°C, well above the T_m_ of PPF‐Li. For PPS‐Li, loss modulus *G*′′ shows no distinct difference after oxidation, whereas storage modulus *G*′ decreases, due to the oxidation‐induced chain scission and a reduction in polymeric entanglement density. In PPF‐Li, by contrast, *G*′ after electrochemical oxidation increases and the complex viscosity becomes less frequency‐dependent, suggesting the formation of local crosslinked domains [35, 36, 37].

In Figure S22, qualitative analysis after electrochemical oxidation was evaluated by FTIR spectroscopy. In the case of PPS–Li, new IR bands emerged at ca. 1580 and ca. 1540 cm^−1^ after electrochemical oxidation. These bands can be assigned to the asymmetric stretching vibrations of carboxylate groups (R–COO^−^–Li^+^), indicating oxidative cleavage and subsequent hydrolysis of the ester linkages in PPS, leading to carboxylates as degradation products. The corresponding symmetric stretching mode of the carboxylates was also observed at ca. 1450 cm^−1^, consistent with literature assignments for metal carboxylates [38, 39]. In contrast, PPF–Li exhibited a suppression of the asymmetric carboxylate stretching bands after electrochemical oxidation compared to PPS–Li. Moreover, the disappearance of the C═C stretching IR band was observed, which is attributed to local crosslinking reactions involving double bonds in PPF. These results provide insights into these observations, revealing that the unsaturated SPEs undergo localized crosslinking through radical cation formation followed by intermolecular coupling under anodic polarization [40, 41, 42]. This partial crosslinking suppresses radical diffusion by restricting chain segmental mobility and mitigates polymer decomposition. As anticipated, oxidative stability was further enhanced through fluorination of the reactive chain ends and photo‐crosslinking [25, 43, 44].

### 2.4 Li Plating/Stripping and Full Cell Cycling Performance

The interfacial reactivity of SPEs against a Li° anode was systematically assessed via galvanostatic plating/stripping cycling in Li°||SPE||Li° symmetric cells at 60°C under a current density of 0.1 mA cm^−^^2^ (Figure 5a,b) based on the electrochemical performance at 60°C summarized in Figure S23 and Table S6. While PPS‐Li and PPF‐Li failed within 170–300 h due to rapid interfacial degradation, symmetric cells with PPM‐Li demonstrated significantly enhanced stability, lasting over 1000 h with only a moderate overpotential rise (∼200 mV). Notably, the fluorinated derivative, PPM‐3F‐Li demonstrated outstanding stability, with an overpotential increase of merely ∼50 mV. As shown in the X‐ray photoelectron spectroscopy (XPS) spectra (Figure S24), PPM‐3F‐Li exhibited a characteristic LiF signal (Li1s ≈ 57 eV and F1s ≈ 684.8 eV), confirming the electrochemical reduction of terminal –CF_3_ groups at the Li surface. The resulting C─F cleavage produces LiF and fluorinated organic fragments, forming a compact LiF‐enriched SEI layer. This interphase facilitates favorable Li^+^ transport kinetics while effectively suppressing parasitic interfacial side‐reactions, thereby ensuring long‐term electrochemical stability [45, 46, 47].

**FIGURE 5:**
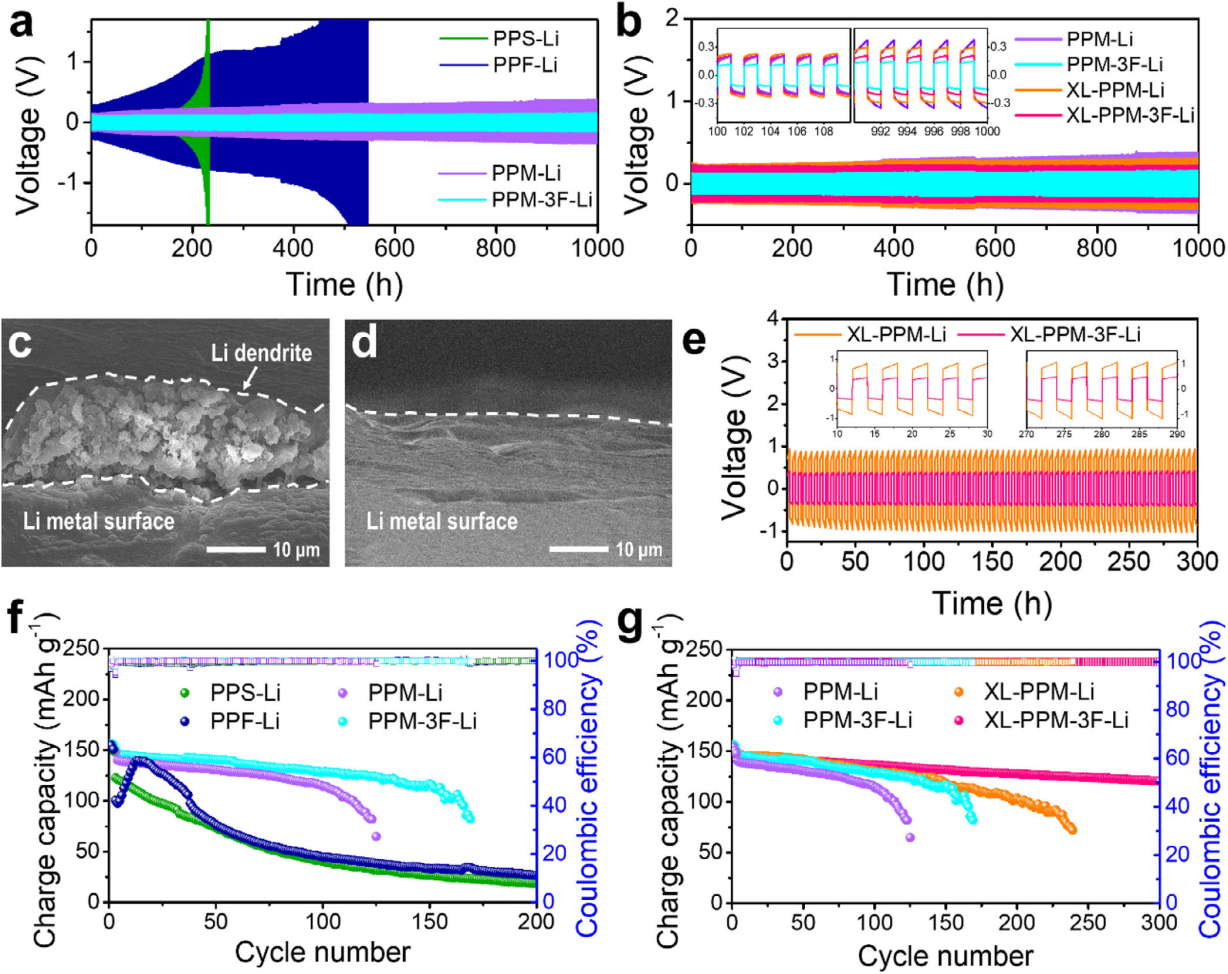
Li plating/stripping cycling performance with (a) uncrosslinked SPEs and (b) PPM derivative‐based SPEs at 0.1 mA cm^−2^ (60°C). Cross‐sectional SEM image of Li° surfaces after Li plating/stripping test for (c) PPS‐Li and (d) XL‐PPM‐3F‐Li. (e) Li plating/stripping cycling performance with XL‐SPEs at 0.4 mA cm^−2^ (60°C). Cycling performance of LFP||SPE||Li° cells using (f) uncrosslinked SPEs and (g) PPM derivative‐based SPEs at 0.3 C (60°C).

Further performance enhancements were realized through photo‐crosslinking. As summarized in Table S4, the Young modulus of XL‐PPM‐Li and XL‐PPM‐3F‐Li increased to 19.2 and 32.0 MPa, respectively, while maintaining comparable ionic conductivity. This mechanical reinforcement physically resists the growth of Li° dendrites during repeated lithium plating/stripping cycling tests, thereby preventing short circuits caused by dendrite penetration. In Figure 5b, after 1000 h of cell operation, the increase in voltage hysteresis for XL‐PPM‐Li was 30 mV lower than that of uncrosslinked PPM‐Li, and remarkably, XL‐PPM‐3F‐Li maintained almost negligible hysteresis growth, approaching zero. Electrochemical impedance spectroscopy (EIS) measurements (Figure S25) corroborated superior interfacial stability of crosslinked SPEs: whereas PPS‐Li and PPF‐Li showed massive increases in interfacial resistances after cycling (25‐ to 400‐fold), XL‐PPM‐Li and XL‐PPM‐3F‐Li exhibited only minimal increases (800–1000 and ∼10 Ω, respectively). As shown in Figure 5c,d, post‐mortem SEM imaging taken after Li plating/stripping cycling clearly reveals the differences in interfacial morphology. PPS‐Li formed irregular, moss‐like dendrites, consistent with unstable Li plating onto a Li° anode surface. Conversely, XL‐PPM‐3F‐Li preserved a uniform, dendrite‐free Li° surface, underscoring the stabilizing effect of photo‐crosslinking combined with –CF_3_ end‐group functionalization [48, 49, 50]. With a further increase in current density up to 0.4 mA cm^−2^ (Figure 5e), XL‐PPM‐3F‐Li still sustained stable Li plating/stripping cycling over 300 h with nearly flat voltage profiles, confirming the robustness of the crosslinked network.

The practical cycling stability of the SPEs was evaluated in LFP||SPE||Li° full cells at 60°C under a C‐rate of 0.3 C as shown in Figure 5f and Figure S26. The cells employing PPS‐Li exhibited a rapid capacity drop, arising from poor interfacial stability against Li metal, which causes non‐uniform Li plating/stripping and accumulation of electrochemically inactive “dead” lithium, which collectively leads to increased interfacial resistance and accelerated capacity loss. PPF‐Li cells also failed prematurely, attributable to their intrinsically low $\sigma_{Li^{+}}$. The abrupt capacity drops and subsequent partial recovery indicate kinetic limitations associated with the semi‐crystalline structure near their *T_m_* (∼66°C). In contrast, PPM‐based SPEs demonstrated markedly improved cycling stability, ascribed to their higher $\sigma_{Li^{+}}$ and superior interfacial compatibility. When further modified via photo‐crosslinking, notably, XL‐PPM‐3F‐Li effectively suppressed Li dendrite formation, enabling intimate and stable interfacial contact and delivering outstanding durability, with capacity retention of 86.6% even after 300 cycles without noticeable capacity fading (Figure 5g). These results clearly establish that the combination of contorted polymer backbone, UV‐induced interchain crosslinking, and fluorinated end‐group chemistry ultimately enables a mechanically robust and highly Li^+^ conductive SPE matrix, capable of sustaining prolonged cycling in practical full‐cell configurations.

### 2.5 Application to Binder‐Electrolyte‐Separator Unified Systems

The XL‐PPM‐3F network, constructed under a precisely controlled crosslinking density, displays well‐balanced mechanical robustness and flexibility along with intrinsic adhesive properties. Based on these material attributes, a new electrode–electrolyte monolith fabrication protocol was developed in which the XL‐SPE simultaneously serves as a binder and a separator, completely replacing conventional PVDF binders. This binder‐free system increases the gravimetric energy density while reinforcing the continuity of the conductive percolation network. At the same time, the insulating binder domains within the electrode are markedly reduced, thereby improving both electronic transport and interfacial kinetics. As shown in Figure 6a, on the calendered electrode that contains conductive carbons and PPM‐3F‐Li, a diluted PPM‐3F‐Li solution containing 1 wt.% of the photo‐initiator was cast, followed by drying and UV‐curing. The resulting interpenetrated electrode–electrolyte monolith, as shown in Figure S27 established an exceptionally well‐integrated interfacial architecture, alleviating the interfacial contact issues in conventional cell configuration where the electrode and XL‐SPE thin films are separately prepared and stacked, which typically accompanies voids at the interface (Figure 6b).

**FIGURE 6:**
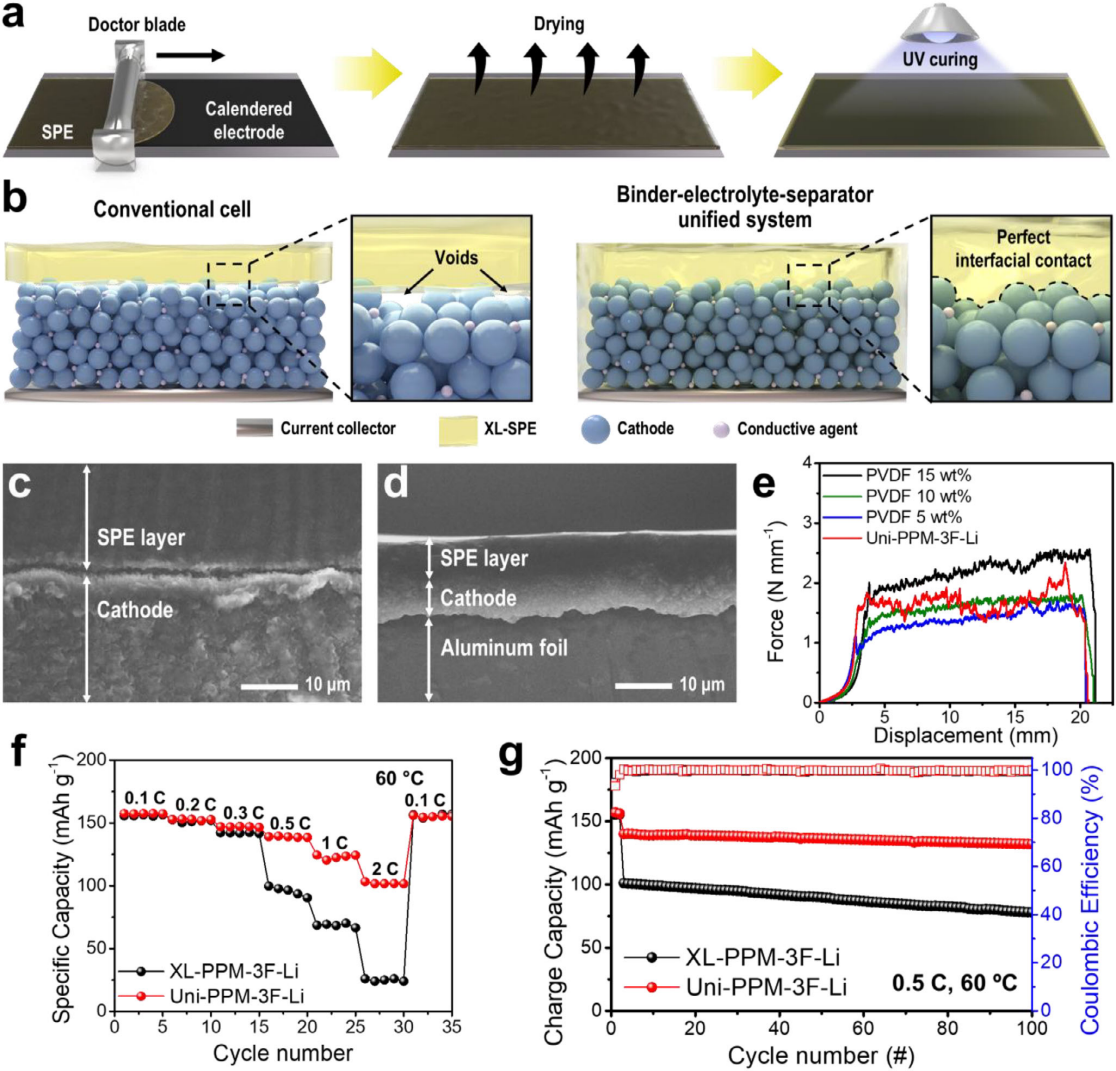
(a) Schematic illustration of the preparation process for the binder‐electrolyte‐separator monolith. (b) Comparison of the electrode–electrolyte interface in conventional cell configurations and binder‐electrolyte‐separator unified systems. Cross‐sectional SEM image of (c) conventional LFP||XL‐PPM‐3F‐Li||Li° cell and (d) LFP||Uni‐PPM‐3F‐Li||Li° cell. (e) 90° peeling strength of the LFP‐Uni‐PPM‐3F‐Li monolith in comparison with conventional electrodes containing different PVDF binder compositions. (f) Rate capability of LFP||XL‐PPM‐3F‐Li||Li° and LFP||Uni‐PPM‐3F‐Li||Li° cells. (g) Cycling performance of LFP||Uni‐PPM‐3F‐Li||Li° and LFP||XL‐PPM‐3F‐Li||Li° cells at 0.5 C.

In Figure 6c, cross‐sectional SEM analyses revealed that the unified monolith features a fully conformal interface, with no observable voids between the electrode components and the cast PPM‐3F‐Li layer. In contrast, in Figure 6d, the conventional cell stack of the electrode and XL‐PPM‐3F‐Li thin films exhibited distinctive voids at the interface. This discrepancy originates from the fact that the polymer chains of the XL‐SPE network are dimensionally fixed after UV‐curing, which hinders wetting and conforming to the electrode microstructure. This results in incomplete and nonuniform interfacial contact. Energy dispersive X‐ray spectroscopy (EDS) mapping of the electrode cross‐sections (Figures S28 and S29) clearly showed that the conventional cell stack with XL‐PPM‐3F‐Li thin film contains ca. 5 atomic% fluorine, originating from both the PVDF binder and the LiTFSI salt in the SPEs. By contrast, the unified monolith exhibited only ca. 3 atomic% fluorine, which is solely attributed to the LiTFSI salt in the SPEs.

To evaluate the adhesion strength of the unified monolith that eliminates PVDF binders, a 90° peel test was conducted (Figure 6e). The conventional LFP electrodes exhibited a peel strength of ca. 1.4 to 2.2 N mm^−1^ with different PVDF compositions (5, 10, and 15 wt.%), whereas the unified monolith utilizing PPM‐3F‐Li (Uni‐PPM‐3F‐Li) showed an adhesion strength of 1.7 N mm^−1^, which is a similar level to that of an electrode with 10 wt.% PVDF. Notably, the unified electrode architecture can be extended to cathodes employing LFP and NCM 622 active materials even at higher areal loadings (Figure S30 and Table S7). When evaluated using identical active materials and areal loadings, the unified electrodes exhibited adhesion strengths comparable to, or slightly higher than, those of conventional PVDF‐based electrodes. Furthermore, surface and interfacial cutting analysis system (SAICAS) depth‐profiling and FTIR spectroscopy verified the uniform formation of a crosslinked network throughout the unified electrode without a crosslinking gradient across the entire thickness, thereby providing augmented cohesion and adhesion over the PVDF binder system (Figure S31).

Taken together, it is confirmed that the unified system enabled by crosslinking SPEs can completely replace any other conventional binders, achieving sufficient interfacial adhesion. The interfacial characteristics of the unified architecture were further examined by EIS analyses in Li metal full‐cell configurations. As shown in Figure S32, the conventional LFP||XL‐PPM‐3F‐Li||Li° cell exhibited an R_sol_ of 447 Ω and an R_ct_ of 465 Ω. In contrast, the Uni‐PPM‐3F‐Li displayed substantially lower impedance values, with an R_sol_ of 340 Ω and an R_ct_ of only 120 Ω, ensuring more efficient ionic transport pathways and charge‐transfer kinetics.

Furthermore, the rate capabilities shown in Figure 6f highlight the performance differences between the two cell configurations. The LFP||XL‐PPM‐3F‐Li||Li° cell retained 91% and 62% of its initial reversible capacity at 0.3 C and 0.5 C, respectively, followed by a severe capacity drop at even higher current densities. This capacity drop at elevated C‐rates can be attributed to pronounced ohmic drop across XL‐SPE owing to its finite ionic conductivity and amplified interfacial polarization under high current density, leading to an earlier reach of the cutoff voltage and thereby limiting the accessible capacity. Particularly, even minor interfacial contact imperfections in the LFP||XL‐PPM‐3F‐Li||Li° cell can be amplified under high current densities, leading to a pronounced capacity drop at high C‐rates, while excellent capacity retention is maintained during prolonged cycling at a moderate current density of 0.3 C. In contrast, the LFP||Uni‐PPM‐3F‐Li||Li° cell exhibited a gradual decrease in discharge capacity as the current density increased from 0.1 C to 2 C, retaining 65% of its capacity even at 2 C. When the rate performance was evaluated at 40°C using LFP electrodes with an areal loading of 5 mg cm^−2^, the performance gap became even more pronounced (Figure S33). Owing to severe kinetic limitations, the LFP||XL‐PPM‐3F‐Li||Li° cell delivered only 53% of the reversible capacity at 0.2 C, and stable capacity could not be achieved at 0.5 C or higher rates. In contrast, the LFP||Uni‐PPM‐3F‐Li||Li° cell exhibited 90%, 79%, and 65% of the reversible capacity at 0.2 C, 0.3 C, and 0.5 C, respectively. These results indicate improved interfacial contact and facilitated ionic/electronic transport enabled by the integrated architecture of the unified system.

Ultimately, the long‐term cycling stability of the Uni‐PPM‐3F‐Li in a Li metal battery was further evaluated under a more demanding condition of 0.5 C at 60°C. Even after 100 cycles, the capacity retention remained highly stable at ∼93.6%, accompanied by an average Coulombic efficiency exceeding 99.8% (Figure 6g and Figure S34). In contrast, the XL‐PPM‐3F‐Li exhibited a pronounced capacity drop immediately after the formation cycles due to the increase of applied current density. After 100 cycles, the capacity retention of the LFP||XL‐PPM‐3F‐Li||Li° cell was only 78%. As shown in Figure S34b and Table S8, the LFP|| XL‐PPM‐3F‐Li||Li° cell showed an R_ct_ of ∼480 Ω and R_SEI_ of 389 Ω after 100 cycles, while the LFP||Uni‐PPM‐3F‐Li||Li° cell demonstrated a substantially lower R_ct_ of ∼238 Ω and R_SEI_ of ∼267 Ω, attributed to significantly improved interfacial charge‐transfer kinetics. Additionally, Uni‐PPM‐3F‐Li was further evaluated under high–areal‐loading conditions (Figure S35). When the LFP||Uni‐PPM‐3F‐Li||Li° cell with an areal loading of 5 mg cm^−^^2^ was cycled at 60°C, an initial reversible capacity of 160 mAh g^−^^1^ was observed during the formation cycle, followed by an initial reversible capacity of 134 mAh g^−^^1^ at a 0.5 C rate, with 93.8% of this capacity retained after 100 cycles. Similarly, when NCM 622 electrodes were employed, the NCM||Uni‐PPM‐3F‐Li||Li° cell exhibited initial reversible capacity of 191 and 168 mAh g^−1^ at a 0.5 C rate, retaining 80.3% of this capacity after 100 cycles under identical conditions. In comparison, the NCM||XL‐PPM‐3F‐Li||Li° cell exhibited a lower reversible capacity of 140 mAh g^−^^1^ at a 0.5 C rate and inferior capacity retention of 61.4% after 100 cycles, which is attributed to increased interfacial resistance arising from poorer interfacial contact and a much thicker electrolyte layer compared to the unified system (Figure S36). Taken together, the combination of favorable rate capability and sustained capacity retention behavior of the Uni‐PPM‐3F‐Li highlights the strong potential of this crosslinking‐enabled binder–separator–electrolyte unified system for ASSLMB.

## 3 Conclusion

We present synergistic strategies to achieve favorable Li^+^ solvation environments and the consequent SPE performance in terms of molecular geometry (*cis*/*trans* stereoisomerism), polymer conformation, crosslinking, and end‐group functionality. Particularly, the *cis*‐configuration of PPM induces a more contorted polymer chain structure, which generates greater free volume within the polymeric host matrix for lithium salts compared to its *trans*‐analog, PPF. This structural feature, coupled with improved Li^+^–TFSI^−^ dissociation energetics, facilitates efficient Li^+^ transport. Furthermore, the distorted conformation of PPM disrupted its interchain packing, yielding a sparser and regulated crosslinked network upon UV‐curing. This achieved an optimal balance between high ionic mobility and sufficient Young modulus, which effectively mitigated Li° dendrite formation. Additionally, end‐group modification contributed to modulating the Li^+^ solvation environment by further lowering the Li^+^–TFSI^−^ coordination/dissociation energy, while improving interfacial stability by promoting the formation of LiF‐rich SEI on the Li° anode. Importantly, the molecular‐level advantages of the designed SPEs were translated into device‐level innovations through a binder–electrolyte–separator unified architecture enabled by an ultrathin XL‐SPE film, which conformally integrated with the electrode microstructure. By eliminating insulating binder domains and the use of conventional porous separators, this unified system markedly reduces interfacial and charge‐transfer resistance, unlocking exceptional high‐rate capability and long‐term cycling stability. The fabricated thin, conformal, and mechanically coherent electrode–electrolyte monolith unfolded in this work represents a fundamental departure from conventional multilayer cell configurations. Collectively, these findings establish how stereochemistry‐driven chain conformation governs the intrinsic trade‐off between ion transport and mechanical strength of crosslinked polyester‐based SPEs, while suggesting the application of such optimized crosslinkable SPEs to a binder‐electrolyte‐separator unified system as key enablers for next‐generation ASSLMBs.

## Conflicts of Interest

The authors declare no conflicts of interest.

## Supporting information




**Supporting File**: advs77263‐sup‐0001‐SuppMat.docx.

## Data Availability

The data that support the findings of this study are available from the corresponding author upon reasonable request.
