# Supplementary material for: Tailored Stereo‐Configuration of Crosslinkable Polyester‐Based Solid Polymer Electrolytes for All‐Solid‐State and Binder‐Free Li Metal Batteries
Source: Adv Sci (Weinh). 2026 Aug 24;13(63):e00063. doi: 10.1002/advs.202600063 (PMC13502266; doi:10.1002/advs.202600063)
Supplement: Supplementary file 1 [file ADVS-13-e00063-s001.docx]

Supporting Information

**Tailored Stereo-Configuration of Crosslinkable Polyester-Based Solid Polymer Electrolytes for All-Solid-State and Binder-Free Li Metal Batteries**

*Hyun Jun Song, Jeong Yoon Kim, Sungpyo Hong, Yong Hui Kim, Myeong Gyun Nam, Jeong-Won Ho, Min Gi Kang, Hye Seon Kim, Chaeyeon Ha, Sang Uck Lee*, Pil J. Yoo*, and Patrice Rannou**

H. J. Song, J. Y. Kim, S. Hong, Y. H. Kim, M. G. Nam, J.-W. Ho, S. U. Lee, M. G. Kang, H. S. Kim, P. J. Yoo
School of Chemical Engineering, Sungkyunkwan University (SKKU), Suwon 16419, Republic of Korea
E-mail: [suleechem@skku.edu](mailto:suleechem@skku.edu) ; [pjyoo@skku.edu](mailto:pjyoo@skku.edu)

C. Ha, P.J. Yoo
SKKU Advanced Institute of Nanotechnology (SAINT), Sungkyunkwan University (SKKU), Suwon 16419, Republic of Korea
P.J. Yoo
SKKU national lab for Intelligent Energy Solution Technology (SIEST), Sungkyunkwan University (SKKU), Suwon 16419, Republic of Korea
P. Rannou
Univ. Grenoble Alpes, Université Savoie Mont-Blanc, CNRS, Grenoble-INP, LEPMI, 38000 Grenoble, France
Email: [patrice.rannou@grenoble-inp.fr](mailto:patrice.rannou@grenoble-inp.fr)

**Index**

**Scheme S1.** Synthetic pathways toward a) poly(*n*-pentyl succinate), b) poly(*n*-pentyl fumarate), and c) poly(*n*-pentyl maleate) via a tetraisopropyl orthotitanate-catalyzed (0.01 eq. vs. 1.00 eq. of a dimethyl ester) transesterification A_2_B_2_ polycondensation route.

**Scheme S2.** Synthetic pathway toward the OH reactive end-group modification of the hemitelechelic PPM using trifluoroacetic anhydride.

**Table S1.** O:Li ratios (*r =* [O]/[Li]) for PPS-Li, PPF-Li, PPM-Li, and PPM-3F-Li as a function of LiTFSI content (20-40 wt%)

**Scheme S3.** Equivalent electrical circuit used to model the impedance of SS||SPE||SS symmetric cells.

**Figure S1.** a) ^19^F NMR spectra of TFT, TFT+PPM and TFT+PPM-3F. b) Peak integrations in ^19^F NMR spectra of PPM-3F for end-group conversion calculation.

**Figure S2.** ^19^F NMR spectra of PPM-3F (black) vs. PPM-3F-Li (red) with a LiTFSI (*r =* 18).

**Figure S3.** TGA thermograms of poly(*n*-alkyl esters) a) without (host matrices) and b) with LiTFSI (*r =* 18) (SPEs).

**Table S2.** SEC and NMR analyses of the host polymer matrices: weight-average molar mass (M_w_), number-average molar mass (M_n_ from SEC and NMR), dispersity in weight (D_w_=M_w_/M_n_), and number-average degree of polymerization (DP_n_)

**Figure S4.** ^1^H and ^13^C NMR spectra and chemical structures of a) PPS, b) PPF, c) PPM, and d) PPM-3F.

**Figure S5.** Peak integrations in ^1^H NMR spectra of a) PPS, b) PPF and c) PPM for number-average molecular weight (M_n_) calculation by end-group analysis.

**Figure S6.** Overlayed SEC traces of PPS, PPF, PPM, and PPM-3F.

**Figure R7.** ^1^H NMR spectra of a) pristine PPM and b) the isomerized product (PPF). c) Overlayed SEC traces of pristine PPM and the isomerized product (PPF).

**Figure S8.** DSC Thermograms of poly(*n*-alkyl esters) a) without (host matrices) and b) with LiTFSI (*r =* 18) (SPEs).

**Figure S9.** DSC analysis for PPF and PPF-Li.

**Figure S10.** Photographs of synthesized polymer host matrices, SPEs, and XL-SPE thin films. a) PPS, b) PPF, c) PPM, d) PPM-3F, e) XL-PPF, f) XL-PPM, and g) XL-PPM-3F.

**Table S3.** Crosslinking content of the crosslinked polymer matrix thin films

**Table S4.** Young modulus values of the XL-SPE thin films

**Figure S11.** Molecular dynamics (MD) simulation models of polyester-based SPEs. The O:Li ratio of 18:1 corresponds to a 25 wt% in experiments.

**Figure S12.** Mean square displacement (MSD) and diffusivity of Li^+^ cations in polymeric host matrices at 80 °C.

**Table S5.** Calculated diffusion coefficients and relative diffusivity of Li^+^ cations in polymeric host matrices

**Figure S13.** Possible positions of Li⁺ cations and TFSI⁻ anions due to dissociation caused by PPM and PPM-3F hosts matrices.

**Figure S14.** VFT plots of the ionic conductivity for the SPEs of a) PPS-Li, b) PPF-Li, c) PPM-Li, d) PPM-3F-Li, e) XL-PPM-Li, and f) XL-PPM-3F-Li as a function of O:Li ratio and temperature. The *T_g_* values of SPEs used to calculate *1000/(T-T_g_)* were extracted from the DSC results in Figure 2d.

**Figure S15.** VFT plots of Li^+^ conductivity for the SPEs of a) PPS-Li, b) PPF-Li, c) PPM-Li, d) PPM-3F-Li, e) XL-PPM-Li, and f) XL-PPM-3F-Li as a function of O:Li ratio and temperature. The *T_g_* values of SPEs used to calculate *1000/(T-T_g_)* were extracted from the DSC results in Figure 2d.

**Figure S16.** Li^+^ cationic transference number ($t_{Li}^{+}$) of SPEs employing a) PPS-Li, b) PPF-Li, c) PPM-Li, d) PPM-3F-Li, e) XL-PPM-Li, and f) XL-PPM-3F-Li as a function of O:Li ratio and temperature.

**Figure S17.** Chronoamperometry (CA) curves and EIS Nyquist diagrams (with magnified graph) before and after polarization at 80 °C using Li^°^||SPE||Li^°^ symmetric cells with SPEs of a) PPS-Li, b) PPF-Li, c) PPM-Li, d) PPM-3F-Li, e) XL-PPM-Li, and f) XL-PPM-3F-Li (*r =* 18).

**Figure S18.** Gradient-dependent PFG NMR signal attenuation profiles in PPM-3F and XL-PPM-3F SPEs. (a) 7Li for PPM-3F SPE, (b) 19F for PPM-3F SPE, (c)7Li for XL-PPM-3F SPE, and (d) 19F for XL-PPM-3F SPE. Red lines represent the fitted attenuation curves used to extract $D_{\mathrm{Li}^{+}}$and $D_{\mathrm{TFSI}^{-}}$.

**Figure S19.** a) VFT plots of ionic conductivity and Li^+^ conductivity for PEO_400k_-Li (*r =* 18) SPEs using SS||SPE||SS symmetric cells and b) CA curves and EIS Nyquist diagrams before and after polarization at 80 °C using Li^°^||SPE||Li^°^ symmetric cells with PEO_400k_-Li SPEs. c) LSV curves for PEO_400k_-Li SPEs at 30 °C, 60 °C and 80 °C.

**Figure S20.** Magnified LSV curve for all 6 SPEs at a) 25 °C and b) 80 °C.

**Figure** **S21.** Storage modulus (*G′*) loss modulus (*G′′*) and complex viscosity (*η**) of a) PPS-Li and c) PPF-Li (*r* = 18) before 10 repeated LSV cycles and b) PPS-Li and d) PPF-Li after 10 repeated LSV cycles.

**Figure S22.** FTIR spectra of a) PPS-Li and b) PPF-Li (*r* = 18) before and after 10 cycles of LSV.

**Figure S23.** LSV curves of the SPEs at 60 °C.

**Table S6.** Ionic conductivity and lithium conductivity of all SPEs at 60 °C

**Figure S24.** a) Li1s, b) F1s, c) C1s, and d) O1s XPS spectra of the Li° surface before and after 20 cycles of Li plating/stripping test employing PPM-Li and PPM-3F-Li.

**Figure S25.** Nyquist plots for Li°||SPE||Li° symmetric cells a), c) before and b), d) after Li plating/stripping cycling test at 0.1 mA cm^-2^.

**Figure S26.** Galvanostatic charge-discharge profile of LFP॥SPE॥Li° cells (0.3 C, 60 °C) using SPEs of a) PPS-Li, b) PPF-Li, c) PPM-Li, d) PPM-3F-Li, e) XL-PPM-Li, and f) XL-PPPM-3F-Li.

**Figure S27.** Photographs of LFP cathode-Uni-PPM-3F-Li monolith.

**Figure S28.** Cross-sectional SEM image and EDS mapping (Fe, C, and F) of the electrode-electrolyte interface in a) conventional cell stack of LFP cathode and XL-PPM-3F-Li and b) Uni-PPM-3F-Li monolith.

**Figure S29.** Cross-sectional SEM image, EDS mapping (Fe, C, and F), and distribution atomic ratios of a) conventional cell stack of LFP cathode and XL-PPM-3F-Li and b) Uni-PPM-3F-Li monolith.

**Figure S30.** 90º Peel strength of cathodes with different loading levels: a) LFP-based electrodes and b) NCM-based electrodes. “–uni” denotes the binder–electrolyte–separator unified system, while “–PVDF” represents a conventional electrode containing PVDF-binder.

**Table S7.** Average adhesion strength of 90º peel test with different active materials and areal loading levels

**Figure S31.** Depth-resolved cohesion characterization of NCM-PVDF and NCM-uni electrodes. SAICAS depth profiles of a) NCM-PVDF (with thickness of ~36 μm and areal mass loading of 5 mg cm^-2^) and b) NCM-uni electrodes (with thickness of ~20 μm and areal mass loading of 5 mg cm^-2^), showing horizontal load (black) and vertical load (red) as a function of cutting time, with the corresponding cutting depth (blue) plotted on the right axis. c) ATR-FTIR spectra of NCM-PVDF (black), and the front (blue) and back (red) surfaces of the NCM-uni electrode.

**Figure S32.** Electrochemical impedance spectroscopy characterizations of LFP॥XL-PPM-3F-Li॥Li° cells and LFP॥Uni-PPM-3F-Li॥Li° cells: Nyquist plots.

**Figure S33.** Rate capability of a) LFP||XL-PPM-3F-Li||Li° cell and LFP||Uni-PPM-3F-Li||Li° cell employing higher areal loading levels of 5mg cm^-2^ (40 °C). Galvanostatic charge-discharge profile of b) LFP||XL-PPM-3F-Li||Li° cell and c) LFP||Uni-PPM-3F-Li||Li° cell for each C-rate.

**Figure S34.** a) Galvanostatic charge-discharge profile of LFP॥Uni-PPM-3F-Li॥Li° cell cycling at 0.5 C, 60 °C. b) Electrochemical impedance spectroscopy of LFP॥XL-PPM-3F-Li॥Li° cells and LFP॥Uni-PPM-3F-Li॥Li° cells after 100 cycles and corresponding equivalent circuit.

**Table S8.** R_s_, R_SEI_, and R_ct_ in EIS spectra in **Figure S34b**

**Figure S35.** a) Cycling performance at of LFP||Uni-PPM-3F-Li||Li° and NCM||Uni-PPM-3F-Li||Li° employing higher areal loading levels of 5 mg cm^-2^ (0.5 C, 60 °C). Galvanostatic charge-discharge profile of b) LFP॥Uni-PPM-3F-Li॥Li° cells, and c) NCM॥Uni-PPM-3F-Li॥Li° cells.

**Figure S36.** a) Cycling performance of NCM||XL-PPM-3F-Li||Li° and NCM||Uni-PPM-3F-Li||Li° cells employing higher areal loading levels of 5 mg cm^-2^ (0.5 C, 60 °C). Galvanostatic charge-discharge profile of b) NCM॥XL-PPM-3F-Li॥Li° cells, and c) NCM॥Uni-PPM-3F-Li॥Li° cells.

**Table S9.** Performance comparison of polyester-based SPEs

**Experimental section**

**Materials**

1,5-pentanediol (>97.0%, TCI), Dimethyl succinate (>98.0%, TCI), Dimethyl fumarate (>98.0%, TCI), Dimethyl maleate (>97.0%, TCI), Tetraisopropyl orthotitanate (TCI), Trifluoroacetic anhydride (≥99%, Sigma-Aldrich), Phenylbis(2,4,6-trimethylbenzoyl)phosphine oxide (Irgacure® 819, Sigma Aldrich), Bis(trifluoromethane sulfonyl)imide lithium salt (LiTFSI, 99.95%, Sigma-Aldrich), Lithium iron phosphate (LFP, Wellcos),  Lithium nickel manganese cobalt oxide (NCM, LiNi_0.65_Co_0.15_Mn_0.2_O_2,_ Wellcos) Super P (Wellcos), Poly(vinylidene fluoride) (PVDF, Sigma Aldrich M_w_=180 kDa), 1-methyl-2-pyrrolidinone (NMP, Sigma Aldrich, 99.5%), Chloroform (≥99.5%, Sigma-Aldrich), Diethyl ether (Sigma Aldrich, 99%), anhydrous Tetrahydrofuran (≥99.9%, Sigma-Aldrich), and Poly(ethylene glycol) methyl ether (mPEG, Mn=~2,000, Sigma-Aldrich) were used as received, unless otherwise stated.

**Syntheses of poly(*n*-alkyl esters)**

**Scheme S1.** Synthetic pathways toward a) poly(*n*-pentyl succinate), b) poly(*n*-pentyl fumarate), and c) poly(*n*-pentyl maleate) via a tetraisopropyl orthotitanate-catalyzed (0.01 eq. vs. 1.00 eq. of a dimethyl ester) transesterification A_2_B_2_ polycondensation route.

The three different poly(*n*-alkyl esters), poly(*n*-pentyl succinate) (PPS), poly(*n*-pentyl fumarate) (PPF), and poly(n-pentyl maleate) (PPM), were synthesized via a transesterification A_2_B_2_ polycondensation condensation route between dimethyl succinate, dimethyl fumarate, and dimethyl maleate (dimethyl esters) and 1,5-pentanediol. In a typical procedure, 1,5-pentanediol (200 mmol) and the dimethyl ester (220 mmol) were mixed in a 1:1 molar feed ratio and stirred in a 250 mL three-necked flask at 120 °C for 30 min under an argon (Ar) flow. The flask was equipped with a reflux condenser to avoid the evaporation of the reactants. After 30 min of stirring, tetraisopropyl orthotitanate (2 mmol, 0.01 equivalent with respect to dimethyl esters) was slowly added into the reactor, the reaction medium being additionally stirred at 120 °C for 12 h. Then the reactor was first kept under a moderate dynamic vacuum (base pressure of *ca.* 200 mbar) at 120 °C for a period of 3 h, followed by a final stage under a higher dynamic vacuum (base pressure of *ca*. 0.1 mbar) at 150 °C for 3 h. The resulting pale-yellow viscous polymer was cooled down to room temperature, dissolved in chloroform, and precipitated in cold (*ca*. 6 °C) diethyl ether. The precipitate was finally dried at 120 °C under a primary dynamic vacuum (base pressure of 0.01 mbar) for 12 h to remove traces of residual solvent.

**Synthesis of PPM-3F**

**Scheme S2.** Synthetic pathway toward the OH reactive end-group modification of the hemitelechelic PPM using trifluoroacetic anhydride.

The dried hemitelechelic PPM was dissolved in anhydrous THF, placed in a 250 mL three-necked flask under Ar flow. After 30 min of Ar purging, trifluoroacetic anhydride (0.6 molar equivalent of PPM) was added to the batch dropwise and stirred at 0 °C (in ice bath) for 1 hour. Then, the reaction medium was stirred at 25 °C and 55 °C for 6 h and 2 h, respectively. Finally, PPM-3F was collected after precipitation in cold (ca. 6 °C) diethyl ether and vacuum dried at 120 °C under a primary dynamic vacuum (base pressure of *ca.* 0.01 mbar) for 12 h.

**Solid Polymer Electrolyte (SPE) preparation**

The polymer host matrices and LiTFSI were respectively dissolved in chloroform and THF stock solutions, and mixed according to the desired salt concentration (20 to 40 wt%. Note that 25 wt% corresponds to a O:Li molar ratio (*r* = [O]/[Li]) of 18:1). The mixtures were stirred at room temperature for 1 hour. After the removal of the solvent medium by a rotavapor, the SPEs were vacuum-dried at 120 °C for 12 h under a primary dynamic vacuum (base pressure of ca. 0.01 mbar) to result in quasi-solid-state polymer electrolytes. The resulting Li-containing SPEs are referred to as Polymer-Li.

**Table S1.** O:Li ratios (*r =* [O]/[Li]) for PPS-Li, PPF-Li, PPM-Li, and PPM-3F-Li as a function of LiTFSI content (20-40 wt%)

|  | 20 wt% | 25 wt% | 30 wt% | 35 wt% | 40 wt% |
| --- | --- | --- | --- | --- | --- |
| PPS-Li | 24:1 | 18:1 | 14:1 | 11:1 | 9:1 |
| PPF-Li | 24:1 | 18:1 | 14:1 | 11:1 | 9:1 |
| PPM-Li | 24:1 | 18:1 | 14:1 | 11:1 | 9:1 |
| PPM-3F-Li | 24:1 | 18:1 | 14:1 | 11:1 | 9:1 |

**Crosslinked SPE preparation**

Irgacure® 819 (1 wt%) was blended with the SPE to act as a photoinitiator. The mixture was poured into a PTFE mold (ID 18 mm, thickness 200 μm) and exposed to a UV lamp (λ = 365 nm, 15 mW cm^-2^ at a UV Lamp-to-Sample distance of *ca.* 10 cm) for 1 h. The UV crosslinking process was carried out at 100 °C onto a hot plate to keep the polymer chains in a rubbery state while curing. After 1 hour, the free-standing XL-SPE thin films were detached from the mold, dried in a vacuum oven at 100 °C under a primary dynamic vacuum (base pressure of ca. 0.01 mbar) to remove residual moisture for 12 h, and transferred into a glove box for cell assembly.

**Chemical, Physicochemical, Thermal, and Thermomechanical characterization**

^1^H, ^13^C, and ^19^F nuclear magnetic resonance (NMR) spectra were recorded on a Bruker AVANCE III HD 700 spectrometer (700 MHz), using CDCl_3_ as a deuterated solvent. A Bruker AVANCE III HD (500 MHz) was used to record solid-state ^13^C NMR spectra. Size exclusion chromatography (SEC) analyses were carried out on an Agilent 1260 Infinity II system using THF as eluent (PLgel Mixed A/B column set, Agilent; RI detector, RID1; THF eluent, 1.0 mL min^-1^; 40 °C; narrow-dispersity PS standards with weight-average molar masses spanning over the 152–5.97 × 10⁶ g mol^-1^ range). Differential scanning calorimetry (DSC) analyses were carried out using an Hitachi Nexta DSC 600 instrument under the following conditions: 25 °C to 160 °C at a heating rate of 10 °C min^-1^ (1^st^ heating), 160 °C to –80 °C at a cooling rate of 20 °C min^-1^ (1^st^ cooling), –80 °C to 160 °C at a heating rate of 10 °C min^-1^ (2^nd^ heating). Data from the 2^nd^ heating scan were selected and analyzed to extract the glass transition temperatures (*T_g_*). Thermogravimetric analysis (TGA) curves were obtained using a Hitachi STA7200RV apparatus to determine the decomposition temperature (*T_dec_*). After being held at 120 °C for 1 h to remove initial moisture, the measurement was carried out under an ambient atmosphere with a heating rate of 10 °C min^-1^. The weight of each sample loaded into the DSC pan was adjusted to *ca.* 5 mg. The decomposition temperature was determined from the maximum weight loss rate in the DTG curve. Fourier transform infrared (FT-IR) spectroscopy in the attenuated total reflectance (ATR) mode was conducted on neat materials using a Shimadzu IRTracer-100. The tensile strength of XL-SPE films was measured by tensile tests using a universal testing machine (UTM, 3343, Instron). The rheological properties of the SPEs were investigated using a TA ARES-G2 rheometer at 80 °C. The adhesion strength of the electrodes was evaluated using a 90° peel test with UTM. The electrode samples were mounted on a universal testing machine, and the peel force was measured at a constant crosshead speed of 10 mm min^-1^. The average peel strength was calculated from the steady-state peeling region of the force–displacement curve. The surface and interfacial cutting analysis system (SAICAS) depth-profiling was optained by cutting the electrode samples with BN blade (Width=1 mm) at a shearing angle of 45° and constant velocity (horizontal speed=3㎛/sec, vertical speed=0.3㎛/sec), using a DN-EX (DAIPLA WINTES CO., LTD).

**Coin cell assembly and electrochemical characterizations**

All electrochemical characterizations were carried out using a Bio-Logic VSP potentiostat. To evaluate ionic conductivity, the SPEs were sandwiched between two (ion-blocking) stainless steel (SS) electrodes to generate SS||SPE||SS symmetric cells in a glove box (0.1 ppm of O_2_ and 0.1 ppm of H_2_O). A PTFE thin film (outer diameter (OD)=19 mm; inner diameter (ID)=12 mm; thickness=200 µm) was used as a separator for PPS, PPF, and PPM-based SPEs loaded into the punched hole. This PTFE seperator was employed to contain the electrolyte and ensure a stable thickness of the SPE layer, eliminating the risk of internal short circuits during cycling arising from decreased viscosity of SPEs at elevated temperature. For the XL-SPEs thin films, the crosslinked membranes were directly sandwiched in between the SS electrodes. The electrochemical impedance spectra were recorded in the 1.0 MHz-0.1 Hz frequency range with an amplitude of 10 mV, scanned from 30 °C to 80 °C. The bulk ionic conductivity (*σ*) was calculated by the following equation:

$$\begin{aligned} \sigma=\frac{L}{R_{b}\times A} \#\left( 1 \right) \end{aligned}$$

where *L* is the electrolyte thickness, *A* is the active area of the electrode surface, and *R_b_* is the bulk resistance extracted from the Nyquist plots. The Equivalent electrical circuit for SS||SPE||SS symmetric cells is expressed in **Scheme S3**, where R_b_ is the bulk resistance, C_1_ is the geometric capacitance, and C_d_ is the double layer capacitance [1].

**Scheme S3.** Equivalent electrical circuit used to model the impedance of SS||SPE||SS symmetric cells.

The temperature-dependent ionic conductivity (*σ*) was analyzed through a Vogel–Fulcher-Tammann (VFT) expressed as follows:

$\begin{aligned} \sigma=A exp\left[ -\frac{B}{T-T_{g}} \right] \#\left( 2 \right) \end{aligned}$where *A* is the pre-exponential factor related to the number of charge carriers in the electrolyte, *B* is the parameter related to the activation energy barrier for ion transport, *T* is the absolute temperature, and *T_g_* is the glass transition temperature. The linear conductivity–temperature relationship was depicted by the plot of *logσ* vs. *1000/(T-T_g_)* [2].

To evaluate the Li^+^ cationic transference number ($t_{Li}^{+}$), the SPEs were sandwiched between two Lithium metal (Li°) foils backed with SS spacers to yield Li°||SPE||Li° symmetric cells. Electrochemical impedance spectra were recorded before and after chronoamperometry (CA) to determine $t_{Li}^{+}$ through the modified Bruce-Vincent method:

$$\begin{aligned} t_{Li}^{+}=\frac{i_{SS}}{i_{O}} \frac{\Delta V - i_{O}R_{CT}^{O}}{\Delta V - i_{SS}R_{CT}^{SS}} \#\left( 3 \right) \end{aligned}$$

where *i_0_* and *i_ss_* refer to initial and steady-state current densities in the CA curve, respectively, and *ΔV* is the DC potential (30 mV) applied across the cell. $R_{CT}^{O}$ and $R_{CT}^{SS}$ are the interfacial impedances at initial and steady state, respectively [3, 4].

Consequently, the Li⁺ ionic conductivity ($\sigma_{{Li}^{+}}$), defined as the intrinsic ionic conductivity of Li⁺ cations excluding the contribution from TFSI⁻ anions, can be calculated using the following equation:

$$\begin{aligned} \sigma_{{Li}^{+}}=\sigma\times t_{Li}^{+} \#\left( 4 \right) \end{aligned}$$

To evaluate the electrochemical stability window (ESW), the SPEs were sandwiched between a SS spacer and a Li° foil backed with another SS spacer to results in Li°||SPE||SS asymmetric cells. The linear sweep voltammetry (LSV) curves were recorded up to 6 V (vs. open circuit voltage) at a scan rate of 0.5 mV/s.

**Li stripping/plating test**

To evaluate lithium stripping/plating behavior, Li°||SPE||Li° symmetric cells were assembled using the synthesized SPEs. For viscous SPEs, a 200 μm-thick PTFE thin film was employed as a separator, whereas XL-SPE thin films were directly utilized without any additional separator. The assembled cells were cycled at 60 °C under a moderate current density of 0.1 mA cm⁻² for 1 h per each charge and discharge steps, over a total duration of 1000 h. For high current density measurements, the cells were cycled at 0.4 mA cm^-2^ for 2 h per each charge and discharge steps, over a total of 300 h.

**Post-cycling Analyses**

Electrochemical Impedance Spectroscopy (EIS) measurements before and after lithium stripping/plating (LSP) polarization were conducted using a Bio-Logic VSP potentiostat. The applied voltage amplitude was 1 mV, and the frequency range was swept from 1 MHz to 100 mHz at 60 °C. For morphological observation of the Li° surface, Li°||SPE||Li° symmetric cells after LSP cycling were disassembled in an argon-filled glovebox (0.1 ppm of O_2_ and 0.1 ppm of H_2_O). The Li° electrodes were carefully extracted from the coin cells, rinsed with dimethyl carbonate (DMC) to remove residual SPEs, and subsequently dried under a primary dynamic vacuum (base pressure of 0.1 mbar) during 2 hours at 60 °C. To investigate the nucleation and growth of Lithium dendrites, Li° cross-sectioned samples were prepared by cross-sectional polisher (IB-19530CP, JEOL) and examined by Field-Emission Scanning Electron Microscopy (FE-SEM, Hitachi, S-4800) in a dry room. For X-ray Photoelectron Spectroscopy (XPS) analyses performed on a ESCALAB250Xi (Thermofisher Scientific) spectrometer, Li° electrodes after 50 cycles of LSP were prepared following the above-described disassembly and rinsing procedures.

**Positive electrodes preparation**

The LiFePO_4_ (LFP) and LiNi_0.65_Co_0.15_Mn_0.2_O_2_ (NCM) positive (+) electrodes were fabricated with a weight ratio of active material, Super P conductive carbon, poly(vinylidene fluoride) (PVDF) binder, and SPEs of 80:5:5:10. First, PVDF was dissolved in 1-methyl-2-pyrrolidone (NMP), to prepare a 10 wt% stock solution, into which SPEs were subsequently added to satisfy the above composition ratio. Separately, the active material and Super P were pre-mixed thoroughly at 2000 rpm using a planetary mixer (AR-100, Thinky, Japan) for 1 minute, followed by the gradual addition of the binder/SPE solution. A small amount of NMP was further added to improve the processability, ensuring a homogeneous slurry suitable for doctor blade processing. The prepared slurry was cast onto aluminum foils using a doctor blade. The (+) electrodes were dried in an oven at 120 °C for 1 h to remove residual solvent, and then subjected to a calendaring process (WCRP-MG1015H, Wellcos Corp., Korea). The final (+) electrodes exhibited an aerial loading of *ca.* 2 mg cm^-2^ unless otherwise mentioned.

**Binder-electrolyte-Separator unified electrode preparation.**

For preparing electrodes for binder-electrolyte-separator unified system, LFP or NCM active material, Super P conductive carbon, and SPE (PPM-3F-Li) containing 2 wt% of Irgacure® 819 were mixed at 2000 rpm using a planetary mixer with a weight ratio of 80:5:15. The resulting slurry was cast onto aluminum foils using a doctor blade. The (+) electrodes were dried in an oven at 120 °C for 1 h to remove residual solvent, followed by UV-curing for 2h using LED UV-curing system (AHLED-100, Ahtech LTS, λ = 365 nm, 25 mW cm^-2^ at a UV Lamp-to-Sample distance of ca. 10 cm) to suppress premature skin formation and promoting more homogeneous network formation throughout the electrode thickness [5]. The (+) electrodes exhibited an areal loading of *ca.* 1 mg cm^-2^ unless otherwised mentioned. Subsequently, the SPE precursor solution, prepared by dissolving the 20 wt% of PPM-3F-Li in a CHCl_3_/THF mixture (3:1 v/v) was cast directly onto the calendared electrode using doctor blade to ensure complete surface coverage. The coated electrodes were dried to remove solvents, and then subjected to a second UV-curing step for 2 h to form the crosslinked SPE layer.

**LFP||SPE||Li° cell and NCM||SPE||Li° cell assembly and charge-discharge cycling tests**

The fabricated (+) electrodes were punched into 12mm disks, welded onto coin-cell cases, and subsequently dried under a primary dynamic vacuum (bases pressure of ca. 0.1 mbar) at 120 °C for 6 h. The cell assembly was carried out in an argon-filled glovebox (0.1 ppm of O_2_ and 0.1 ppm of H_2_O). A PTFE separator (12 mm hole diameter, 200 μm thickness) was employed to contain the viscous SPEs and ensure a stable thickness of the SPE layer. In contrast, for cells employing XL-SPEs, a defined amount of the uncured SPEs was directly dropped onto the welded electrode surface without any separator, followed by smoothing and curing under UV irradiation for 1 h to obtain the crosslinked SPE thin film membranes. The assembled cells were rested for 24 h prior to testing and then subjected to galvanostatic cycling at 60 °C (WMPG 1000, WonATech, Korea). For the initial formation cycle, charge–discharge tests were performed at 0.05 C within a voltage window of 2.5–3.8 V for LFP electrodes and 2.7–4.2 V for NCM electrodes. From the second cycle onward, the cells were cycled at 0.3 C to 0.5 C for long-term performance evaluation.

**Unified system cell assembly and charge-discharge cycling tests**

The unified electrodes were punched into 12mm disks, and subsequently dried under a primary dynamic vacuum (bases pressure of ca. 0.1 mbar) at 120 °C for 6 h. The cell assembly was carried out in an argon-filled glovebox. For long-term cycling performance, the assembled coin cells were subjected to an initial formation cycle at 0.05 C. After formation, the cells were cycled at a constant charge–discharge rate of 0.5 C for the duration of the test at 60 °C. Rate capability tests were also conducted using assembled coin cells at 60 °C. After cell assembly, all cells underwent a formation step consisting of one charge–discharge cycle at 0.05 C. Subsequently, galvanostatic cycling was performed at sequential C-rates of 0.1 C, 0.2 C, 0.3 C, 0.5 C, 1 C, and 2 C, holding each rate for five consecutive cycles. After completing the high-rate sequence, the C-rate was returned to 0.1 C for an additional five cycles to evaluate capacity recovery.

**Computational detail**

All computational simulations were conducted using the Materials Studio 2024 software package [6]. The detailed composition of each simulated system is detailed in **Figure S11**. The simulation parameters for the polymer electrolytes, describing both bonded and non-bonded interactions, were taken from the condensed-phase optimized molecular potentials for atomistic simulation studies (COMPASSⅢ) force field [7, 8]. To maintain charge neutrality, a partial charge of +0.7e was assigned to each Li^+^ cation, with a corresponding -0.7e charge on each TFSI^-^ anion. This charge scaling was employed to better reproduce experimental observations [9–12]. To further assess the sensitivity to charge scaling factors, different factors of 0.6, 0.7, and 0.8 were applied, while keeping all other simulation and analysis conditions unchanged.

Molecular dynamics (MD) simulations were carried out using the Forcite module [13]. Initial amorphous electrolyte cells for each polymer were constructed with the Amorphous Cell module [14–16]. The resulting amorphous cells were subsequently stabilized through a multi-step protocol comprising geometry optimization, thermal annealing, and isothermal-isobaric (NPT) equilibration. In the geometry optimization step, both the lattice constants and internal atomic positions were fully relaxed until the residual forces converged to below 0.001 kcal·mol^-1^·Å^-1^. The annealing procedure involved cyclic temperature variation between 300 K and 500 K to overcome local energy minima and promote structural relaxation. Thereafter, the systems were equilibrated in the NPT ensemble by annealing for 1 ns at 353 K and 1 atm (1.013 × 10⁻⁴ GPa) to ensure convergence of both density and cell dimensions.

In equilibration, a 10 ns production run was performed in the canonical (NVT) ensemble at 353 K to investigate the transport behavior of Li^+^ cations. A time step of 1 fs was used, and the system temperature was controlled by a Nosé–Hoover thermostat [17, 18]. Long-range electrostatic interactions were treated using the particle-particle article-mesh (PPPM) method, whereas van der Waals interactions were calculated with an atom-based scheme and a real-space cutoff distance of 18.5 Å [19]. The diffusion coefficient (D) of Li⁺ cations was obtained from the mean squared displacement (MSD) according to the Einstein relation. The fractional free volume was quantified using the Connolly surface method with a probe radius of 0.1 nm [20]. In addition, the degree of crosslinking, resulting from bond formation between polymer chains, was evaluated within the Forcite module.

To complement the MD simulations, the dissociation energy of Li^+^ cations from LiTFSI was computed using density functional theory (DFT) calculations in the DMol³ module [21, 22]. All DFT calculations were performed for isolated gas-phase species (e.g., polymer, Li^+^, TFSI^-^) without applying periodic boundary conditions. The Perdew-Burke-Ernzerhof (PBE) exchange-correlation functional within the generalized gradient approximation (GGA), combined with the double-numerical plus polarization (DNP) basis set, was employed. Geometry optimizations were considered converged when the energy tolerance reached 10⁻⁵ Ha, the maximum force was below 0.002 Ha·Å^-1^, and the maximum atomic displacement was less than 0.005 Å. An all-electron treatment was applied to all atoms.

**Figure S1.** a) ^19^F NMR spectra of TFT, TFT+PPM, and TFT+PPM-3F. b) Peak integrations in ^19^F NMR spectra of PPM-3F for end-group conversion calculation.

The conversion rate of terminal hydroxyl groups of PPM was quantitatively evaluated by ¹⁹F NMR spectroscopy using a known molar amount (8 μmol) of an internal standard, namely α,α,α-trifluorotoluene (TFT). For the NMR analysis, 28 mg of PPM-3F (M_n_ = 6535 g mol⁻¹, DP_n_=35, as shown in Table S2) was used. Based on the polymer number-average molar mass, the theoretical molar amount of terminal –OCF₃ groups was calculated to be 4.28 μmol. In the ¹⁹F NMR spectrum in **Figure S1b**, the integral of the TFT signal (62.61 ppm) was normalized to 1, and the corresponding integral of the terminal –OCF₃ signal of PPM-3F (74.97 ppm) was measured to be 0.38. From this ratio, the experimentally determined molar amount of –OCF₃ end groups was calculated to be 3.04 μmol. Accordingly, the conversion rate of the terminal hydroxyl groups was estimated to be approximately 71%, calculated as the ratio between the experimentally determined –OCF₃ content and the theoretical value.

**Figure S2.** ^19^F NMR spectra of PPM-3F (black) vs. PPM-3F-Li (red) with a LiTFSI (*r =* 18).

The ^19^F NMR spectra show a distinct resonance near -75 ppm in PPM-3F, and peak shifts to -78 ppm upon Li salt addition, indicating the coordination of Li^+^ cations with fluorinated end-group in the polymer host matrix.

**Figure S3.** TGA thermograms of poly(*n*-alkyl esters) a) without (host matrices) and b) with LiTFSI (*r =* 18) (SPEs).

| Polymers | M_w_  (kDa eq. PS) | M_n_  (kDa eq. PS) | D_w_  (M_w_/M_n_) | M_n_ (NMR, kg/mol) | DP_n_ |
| --- | --- | --- | --- | --- | --- |
| PPS | 12.16 | 6.00 | 2.03 | 6.17 | ~33 |
| PPF | 8.37 | 6.20 | 1.35 | 6.10 | ~33 |
| PPM | 10.85 | 6.53 | 1.66 | 6.47 | ~35 |
| PPM-3F | 11.85 | 6.66 | 1.78 | 6.54 | ~35 |

**Table S2.** SEC and NMR analyses of the host polymer matrices: weight-average molar mass (M_w_), number-average molar mass (M_n_ from SEC and NMR), dispersity in weight (D_w_=M_w_/M_n_), and number-average degree of polymerization (DP_n_)

All host polymer matrices were deliberately designed and synthesized to possess very similar DP_n_ values, thereby ensuring identical O:Li ratios in the corresponding solid polymer electrolytes and enabling a fair comparison of various physical and physicochemical properties. A comparison between the number-average molar masses obtained from SEC analysis (**Figure S6**) and those determined by NMR analyses (**Figure S5**) reveals that a series of host polymer matrices with similar DP_n_ values was successfully synthesized.

**Figure S4.** ^1^H and ^13^C NMR spectra and chemical structures of a) PPS, b) PPF, c) PPM, and d) PPM-3F.

^1^H and ^13^C NMR spectra of each poly(*n*-alkyl esters) host matrices with peak assignments indexed to the corresponding chemical structures. The signals at 7.25 ppm in ^1^H NMR and 77 ppm in ^13^C NMR originate from the CDCl₃ solvent. The insets in the ^1^H NMR spectra highlight the terminal methoxy and CH₂–OH resonances. Comparison of PPM vs. PPM-3F ^1^H NMR spectra (**Figure S4c** vs**. S4d**) shows that the CH₂–OH signal at 3.63 ppm dissapeared after trifluoroacetate modification, confirming substitution of the terminal hydroxyl groups onto the hemitelechelic polymer chains of PPM.

**Figure S5.** Peak integrations in ^1^H NMR spectra of a) PPS, b) PPF and c) PPM for number-average molar mass (M_n_) calculation by end-group analysis.

Number-average degree of polymerization (DP_n_) calculation

a) PPS: The end-group signals at 3.66 ppm (–OCH₃) and 3.62 ppm (–CH₂OH) were normalized to an integral value of 1 (6A = 1, where A is a proportionality constant). The peak at 2.59 ppm corresponds to four methylene protons between the carbonyl groups in each repeating unit, giving a normalized integral of 22.51. Based on the relation 4nA = 22.51, the DPₙ was calculated to be 33 (decimal truncated).

b) PPF: The end-group signals at 3.77 ppm (–OCH₃) and 3.62 ppm (–CH₂OH) were normalized to 1 (6A = 1). The resonance at 6.81 ppm originates from two olefinic protons in the repeating unit, yielding a normalized integral of 11.02. From 2nA = 11.02, the DPₙ was determined to be 33.

c) PPM: The end-group signals at 3.77 ppm (–OCH₃) and 3.63 ppm (–CH₂OH) were normalized to 1 (6A = 1). The signal at 6.23 ppm corresponds to olefinic protons of the PPM repeating unit, while a minor peak at 6.83 ppm arises from trace PPF isomer. The combined integral (11.68) satisfies 2nA = 11.68, resulting in a DPₙ of 35.

**Figure S6.** Overlayed SEC traces of PPS, PPF, PPM, and PPM-3F.

**Figure S7.** ^1^H NMR spectra of a) pristine PPM and b) the isomerized product (PPF). c) Overlayed SEC traces of pristine PPM and the isomerized product (PPF).

As further validation of the similarity in molecular weight and degree of polymerization between the two stereoisomeric polymers, PPM and PPF, we examined whether the PPF obtained via *cis*/*trans* isomerization of PPM retained the same molecular weight as the original PPM. Briefly, PPM was first dissolved in chloroform, after which diethylamine (0.1 equiv.) was added [23]. The reaction mixture was stirred at room temperature overnight, and the resulting product was precipitated into cold diethyl ether and subsequently dried under vacuum. As shown in **Figure S7b**, characteristic vinyl proton signals corresponding to the C=C group of PPF (6.85 ppm) and that of PPM (6.25 ppm) were both observed in the spectrum of the isomerized product. The presence of the PPM resonance indicates that a fraction of unisomerized PPM remained after the reaction. Based on the integration of these vinyl proton signals, the ratio of PPF to residual PPM was estimated to be approximately 4.3:1. The Mₙ was estimated by end-group analysis, following the same method used in **Figure S5**. The calculated Mₙ values of two polymers were comparable: 4.45 kg/mol for pristine PPM and 4.82 kg/mol for the isomerized product. The slight increase of Mₙ after isomerization is attributed to fractionation during the purification process, where shorter oligomer chains are more likely to be extracted, resulting in an apparent increase in the average molecular weight of the recovered polymer. This is consistent with the SEC results in **Figure S7c** where the isomerized product exhibited a narrower trace and a lower dispersity (D_w_) compared to pristine PPM, indicating the preferential additional removal of low-molecular-weight fractions during the purification step.

**Figure S8.** DSC Thermograms of poly(*n*-alkyl esters) a) without (host matrices) and b) with LiTFSI (*r =* 18) (SPEs).

Glass transition temperature (*T_g_*) of all polymer host matrices and SPEs was determined using iso-enthalpic analyses based on fictive temperature method, as several polymer host matrices and SPEs exhibited an enthalpic overshoot in the region of *T_g_* range [24].

**Figure S9.** DSC analyses for PPF and PPF-Li.

**Figure S10.** Photographs of synthesized polymer host matrices, SPEs, and XL-SPE thin films. a) PPS, b) PPF, c) PPM, d) PPM-3F, e) XL-PPF, f) XL-PPM, and g) XL-PPM-3F.

**Table S3.** Crosslinking content of the crosslinked polymer matrix thin films

| Polymers | Initial weight (g) | Final weight (g) | Crosslinking content (%) |
| --- | --- | --- | --- |
| XL-PPF | 3.555 | 3.480 | 97.89 |
| XL-PPM | 3.342 | 1.921 | 57.49 |
| XL-PPM-3F | 3.730 | 2.243 | 60.10 |

The degree of polymer crosslinking was evaluated by a crosslinking content test. The cured cross-linkable polymers were ground into small pieces and immersed into chloroform and stirred under reflux at 60 °C for 24 h. The uncured polymer chains may have dissolved in chloroform, while fully crosslinked networks swell but remain insoluble. The mixture was then subjected to vacuum filtration with several (at least 3) rinsing steps. The filter paper was dried under vacuum at 60 °C overnight. The dried samples were weighed to determine the insoluble fraction, which was used to evaluate the extent of curing.

**Table S4.** Young modulus values of the XL-SPE thin films

| XL-SPE | Young modulus (MPa) |
| --- | --- |
| XL-PPF-Li | 123.4 |
| XL-PPM-Li | 19.2 |
| XL-PPM-3F-Li | 32.0 |

The XL-SPE (*r =* 18). thin films were prepared using square molds (length: 120 mm, width: 10 mm, thickness: 1 mm) and applied in the tensile test [25]. The XL-SPE films were clamped at both ends on the UTM equipment and stretched at a constant speed of 2 mm/min, at room temperature. The linear elastic regions of the strain-strain curves (**Figure 2f**) were used for the determination of the slope, referred to as the Young modulus.

**Figure S11.** Molecular dynamics (MD) simulation models of polyester-based SPEs. The O:Li ratio of 18:1 corresponds to a 25 wt% in experiments.

**Figure S12.** Mean square displacement (MSD) and diffusivity of Li^+^ cations in polymeric host matrices at 80 °C.

**Table S5.** Calculated diffusion coefficients and relative diffusivity of Li^+^ cations in polymeric host matrices

| 5 ns @ 80 °C | Diffusion coefficient (cm^2^/s) | Relative Diffusivity |
| --- | --- | --- |
| PPS | 4.98 ⨯ 10^-8^ | 1.00 |
| PPF | 1.79 ⨯ 10^-8^ | 0.35 |
| PPM | 6.09 ⨯ 10^-8^ | 1.22 |
| PPM-3F | 7.48 ⨯ 10^-8^ | 1.50 |
| XL-PPM | 4.87 ⨯ 10^-8^ | 0.98 |
| XL-PPM-3F | 5.85 ⨯ 10^-8^ | 1.17 |

**Figure S13.** a) Possible positions of Li⁺ cations and TFSI⁻ anions due to dissociation caused by PPM and PPM-3F hosts matrices. b and c) Sensitivity of Li⁺ diffusion to the ionic charge-scaling factor. b) Li⁺ mean-square displacement and c) corresponding Li⁺ self-diffusion coefficients for PPM and PPM-3F obtained using charge-scaling factors of λ=0.6, 0.7, and 0.8. All other simulation and analysis conditions were kept unchanged.

**Figure S14.** VFT plots of the ionic conductivity for the SPEs of a) PPS-Li, b) PPF-Li, c) PPM-Li, d) PPM-3F-Li, e) XL-PPM-Li, and f) XL-PPM-3F-Li as a function of O:Li ratio and temperature. The *T_g_* values of SPEs used to calculate *1000/(T-T_g_)* were extracted from the DSC results in **Figure 2d**.

**Figure S15.** VFT plots of Li^+^ conductivity for the SPEs of a) PPS-Li, b) PPF-Li, c) PPM-Li, d) PPM-3F-Li, e) XL-PPM-Li, and f) XL-PPM-3F-Li as a function of O:Li ratio and temperature. The *T_g_* values of SPEs used to calculate *1000/(T-T_g_)* were extracted from the DSC results in **Figure 2d**.

**Figure S16.** Li^+^ cationic transference number ($t_{Li}^{+}$) of SPEs employing a) PPS-Li, b) PPF-Li, c) PPM-Li, d) PPM-3F-Li, e) XL-PPM-Li, and f) XL-PPM-3F-Li as a function of O:Li ratio and temperature.

**Figure S17.** Chronoamperometry (CA) curves and EIS Nyquist diagrams (with magnified graph) before and after polarization at 80 °C using Li^°^||SPE||Li^°^ symmetric cells with SPEs of a) PPS-Li, b) PPF-Li, c) PPM-Li, d) PPM-3F-Li, e) XL-PPM-Li, and f) XL-PPM-3F-Li (*r =* 18).

**Figure S18.** Gradient-dependent PFG NMR signal attenuation profiles in PPM-3F and XL-PPM-3F SPEs. (a) 7Li for PPM-3F SPE, (b) 19F for PPM-3F SPE, (c)7Li for XL-PPM-3F SPE, and (d) 19F for XL-PPM-3F SPE. Red lines represent the fitted attenuation curves used to extract $D_{\mathrm{Li}^{+}}$and $D_{\mathrm{TFSI}^{-}}$.

Self-diffusion coefficients of Li^+^ and TFSI^-^ were determined by variable-gradient pulsed-field-gradient (PFG) NMR using the ^7^Li and ^19^F resonances, respectively. All measurements were performed at 60 °C on a liquid-state 600 MHz NMR spectrometer (Avance Neo 600 (Prodigy), Bruker). Samples were loaded into the inner tube of a Wilmad® coaxial insert (complete set; inner tube O.D. × I.D. = 2.97 mm × 1.96 mm), while DMSO-d_6_ was placed in the outer tube for field locking. For the crosslinked samples, the precursor polymer electrolyte was first loaded into the inner tube and then subjected to UV irradiation to induce in-tube crosslinking prior to measurement. Both uncrosslinked and crosslinked samples were analyzed using the same instrumental setup. The diffusion coefficients were obtained by fitting the gradient-dependent attenuation of the integrated NMR signal intensities to the Stejskal–Tanner equation as implemented in the fitting routine [26]:
$\begin{aligned} I=I_{0}exp\left[ -D\left( 2\pi\gamma G_{i}\delta\right)^{2}\left( \Delta-\frac{\delta}{3} \right)\times{10}^{4} \right] \#(5) \end{aligned}$where $I$ is the integrated signal intensity at a given gradient strength$G_{i}$, $I_{0}$ is the signal intensity in the absence of gradient attenuation, $D$ is the self-diffusion coefficient, $\gamma$ is the gyromagnetic ratio, $\delta$ is the gradient pulse duration, and $\Delta$ is the diffusion time.

For ^19^F PFG-NMR, the TFSI^-^ resonance was integrated over the range of -80.2 to -78.6 ppm using 32 gradient increments. The fitting employed $I_{0}$=1.005, $\gamma$=4.008$\times$10^3^ Hz G^-1^ , $\delta$=2.400 ms, and $\Delta$=499.9 ms.

For ^7^Li PFG-NMR, the Li^+^ resonance was integrated over the range of -0.8 to 0.5 ppm using 32 gradient increments. The fitting employed $I_{0}$=1.000 $\gamma$=1.655$\times$10^3^ Hz G^-1^, $\delta$=5.000 ms, and $\Delta$=899.9 ms.

PFG-NMR measurements were carried out to independently probe the self-diffusion behavior of Li⁺ and TFSI⁻ in PPM-3F and XL-PPM-3F. Upon crosslinking, the self-diffusion coefficients of both ionic species decreased markedly, indicating an overall restriction of ion mobility within the crosslinked matrix. Specifically, $D_{{Li}^{+}}$ decreased from 6.113$\times$10^-11^ to 8.066$\times$10^-12^ m^2^ s^-1^ (87% decrease), while $D_{{TFSI}^{-}}$ decreased from 4.526$\times$10^-11^ to 4.578$\times$10^-12^ m^2^ s^-1^ (90% decrease). Notably, the reduction in TFSI^-^ diffusivity was slightly greater than that of Li^+^, leading to an increase in the self-diffusion-based Li^+^ fraction, $D_{{Li}^{+}}/\left( D_{{Li}^{+}}+D_{{TFSI}^{-}} \right)$, from 0.575 for PPM-3F to 0.637 for XL-PPM-3F. Although this ratio is not numerically equivalent to the electrochemically measured Li-ion transference number, it shows the same directional trend as the experimentally observed increase in Li transference number upon crosslinking. These results therefore support that crosslinking suppresses anion motion more strongly than Li^+^ transport, which is consistent with the enhanced Li-selective ion transport in XL-PPM-3F.

**Figure S19.** a) VFT plots of ionic conductivity and Li^+^ conductivity for PEO_400k_-Li (*r =* 18) SPEs using SS||SPE||SS symmetric cells and b) CA curves and EIS Nyquist diagrams before and after polarization at 80 °C using Li^°^||SPE||Li^°^ symmetric cells with PEO_400k_-Li SPEs. c) LSV curves for PEO_400k_-Li SPEs at 30 °C, 60 °C and 80 °C.

Li^+^ conductvity ($\sigma_{{Li}^{+}}$) values of PEO_400k_-Li from **Figure S19a** are 0.061 mS cm^-1^ and 0.125 mS cm^-1^ at 60 °C and 80 °C each. The calculated $t_{Li}^{+}$ value of PEO_400k_-Li from **Figure S19b** is 0.17.

**Figure S20.** Magnified LSV curve for all 6 SPEs at a) 25 °C and b) 80 °C.

The oxidation onset potential was defined as the intersection between the tangent line drawn at the steeply rising current region and the baseline.

**Figure** **S21.** Storage modulus (*G′*) loss modulus (*G′′*) and complex viscosity (*η**) of a) PPS-Li and c) PPF-Li (*r* = 18) before 10 repeated LSV cycles and b) PPS-Li and d) PPF-Li after 10 repeated LSV cycles.

**Figure S22.** FT-IR spectra of a) PPS-Li and b) PPF-Li (*r* = 18) before and after 10 cycles of LSV.

**Figure S23.** LSV curves of the SPEs at 60 °C.

| × 10^-4^ S/cm | PPS-Li | PPF-Li | PPM-Li | PPM-3F-Li | XL-PPM-Li | XL-PPM-3F-Li |
| --- | --- | --- | --- | --- | --- | --- |
| Ionic conductivity | 0.425 | 0.114 | 0.385 | 0.793 | 0.352 | 0.483 |
| Li^+^ conductivity | 0.336 | 0.0686 | 0.294 | 0.602 | 0.297 | 0.357 |

**Table S6.** Ionic conductivity and Li^+^ conductivity values of all SPEs at 60 °C

**Figure S24.** a) Li1s, b) F1s, c) C1s, and d) O1s XPS spectra of the Li° surface before and after 20 cycles of Li plating/stripping test employing PPM-Li and PPM-3F-Li.

The presence of a pronounced LiF signal in PPM-3F-Li is attributed to the preferential decomposition of the polymer-bound terminal –CF₃ groups at the Li metal interface. These fluorinated end groups can undergo reductive C–F bond cleavage under strongly reducing conditions, generating F⁻ species that rapidly react with Li° to form a LiF-rich interphase during the early stage of cycling [27]. In contrast, the decomposition of TFSI^-^ anions of LiTFSI is known to typically proceed more gradually and becomes significant at later stages. Therefore, within the limited cycling duration (20 cycles), the contribution of LiTFSI to LiF formation is expected to be minor compared with that of the polymer-bound –CF₃ end groups. As a result, the interphase formed in PPM-3F-Li is dominated by LiF derived from the terminal –CF₃ moieties, whereas the interphase in PPM–Li mainly consists of polymer-derived carbonate and alkoxide species.

**Figure S25.** Nyquist plots for Li°||SPE||Li° symmetric cells a), c) before and b), d) after Li plating/stripping cycling test at 0.1 mA cm^-2^.

**Figure S26.** Galvanostatic charge-discharge profile of LFP॥SPE॥Li° cells (0.3 C, 60 °C) using SPEs of a) PPS-Li, b) PPF-Li, c) PPM-Li, d) PPM-3F-Li, e) XL-PPM-Li, and f) XL-PPPM-3F-Li.

**Figure S27.** Photographs of LFP cathode-Uni-PPM-3F-Li monolith.

**Figure S28.** Cross-sectional SEM image and EDS mapping (Fe, C, and F) of the electrode-electrolyte interface in a) conventional cell stack of LFP cathode and XL-PPM-3F-Li and b) Uni-PPM-3F-Li monolith.

**Figure S29.** Cross-sectional SEM image, EDS mapping (Fe, C, and F), and distribution atomic ratios of a) conventional cell stack of LFP cathode and XL-PPM-3F-Li and b) Uni-PPM-3F-Li monolith.

**Figure S30.** 90º Peel strength of cathodes with different loading levels: a) LFP-based electrodes and b) NCM-based electrodes. “–uni” denotes the binder–electrolyte–separator unified system, while “–PVDF” represents a conventional electrode containing PVDF-binder.

| Adhesive strength  (N mm^-1^) | 5 mg cm^-2^ | 10 mg cm^-2^ |
| --- | --- | --- |
| LFP-PVDF | 1.06 | 0.68 |
| LFP-uni | 0.86 | 0.75 |
| NCM-PVDF | 1.44 | 1.08 |
| NCM-uni | 1.57 | 1.34 |

**Table S7.** Average adhesion strength of 90º peel test with different active materials and areal loading levels

**Figure S31.** Depth-resolved cohesion characterization of NCM-PVDF and NCM-uni electrodes. SAICAS depth profiles of a) NCM-PVDF (with thickness of ~36 μm and areal mass loading of 5 mg cm^-2^) and b) NCM-uni electrodes (with thickness of ~20 μm and areal mass loading of 5 mg cm^-2^), showing horizontal load (black) and vertical load (red) as a function of cutting time, with the corresponding cutting depth (blue) plotted on the right axis. c) ATR-FTIR spectra of NCM-PVDF (black), and the front (blue) and back (red) surfaces of the NCM-uni electrode.

**Figure S32.** Electrochemical impedance spectroscopy characterizations of LFP॥XL-PPM-3F-Li॥Li° cells and LFP॥Uni-PPM-3F-Li॥Li° cells: Nyquist plots.

**Figure S33.** Rate capability of a) LFP||XL-PPM-3F-Li||Li° cell and LFP||Uni-PPM-3F-Li||Li° cell employing higher areal loading levels of 5mg cm^-2^ (40 °C). Galvanostatic charge-discharge profile of b) LFP||XL-PPM-3F-Li||Li° cell and c) LFP||Uni-PPM-3F-Li||Li° cell for each C-rate.

**Figure S34.** a) Galvanostatic charge-discharge profile of LFP॥Uni-PPM-3F-Li॥Li° cell cycling at 0.5 C, 60 °C. b) Electrochemical impedance spectroscopy of LFP॥XL-PPM-3F-Li॥Li° cells and LFP॥Uni-PPM-3F-Li॥Li° cells after 100 cycles and corresponding equivalent circuit.

**Table S8.** R_s_, R_SEI_, and R_ct_ in EIS spectra in **Figure S34b**

|  | R_s_ | R_SEI_ | R_ct_ |
| --- | --- | --- | --- |
| XL-PPM-3F-Li | 744 Ω | 389 Ω | 480 Ω |
| Uni-PPM-3F-Li | 183 Ω | 267 Ω | 238 Ω |

**Figure S35.** a) Cycling performance at of LFP||Uni-PPM-3F-Li||Li° and NCM||Uni-PPM-3F-Li||Li° employing higher areal loading levels of 5 mg cm^-2^ (0.5 C, 60 °C). Galvanostatic charge-discharge profile of b) LFP॥Uni-PPM-3F-Li॥Li° cells, and c) NCM॥Uni-PPM-3F-Li॥Li° cells.

**Figure S36.** a) Cycling performance of NCM||XL-PPM-3F-Li||Li° and NCM||Uni-PPM-3F-Li||Li° cells employing higher areal loading levels of 5 mg cm^-2^ (0.5 C, 60 °C). Galvanostatic charge-discharge profile of b) NCM॥XL-PPM-3F-Li॥Li° cells, and c) NCM॥Uni-PPM-3F-Li॥Li° cells.

**Table S9.** Performance comparison of polyester-based SPEs

| **Polymer electrolyte** | **σ (S cm^-1^) @ Temp.** | **Cathode** | **Cycle No.** | **Capacity retention (%) @ C-rate / Temp.** | **Ref.** |
| --- | --- | --- | --- | --- | --- |
| C5-POE-F/LiTFSI | ~ 1.4ⅹ10^-4^ @ 60 °C | NCM622 | 200 | 70.2 @ 0.1 C / 45 °C | [28] |
| C6-C5-3F/LiTFSI | 5.9ⅹ10^-5^ @ RT | LMFP | 300 | 95 @ 0.2 C / 30 °C | [27] |
| C2-C9-3F/LiTFSI | 2.1ⅹ10^-5^ @ RT | NCM811 | 200 | 74.2 @ 0.2 C / 40 °C | [27] |
| PCL/LiTFSI | 2.1ⅹ10^-5^ @ RT | NCM622 | 100 | 81.6 @ 0.1 C / 55 °C | [29] |
| PEO-PMA double layer/LiTFSI | 2.05ⅹ10^-4^ @ 65 °C | LCO | 100 | 91.2 @ 0.2 C / 65 °C | [30] |
| XL-PPM-3F-Li | 4.63ⅹ10^-5^ @ 60 °C | LFP | 300 | 86.6 @ 0.3 C / 60 °C | **This work** |
| Uni-PPM-3F-Li | - | LFP | 100 | 93.6 @ 0.5 C / 60 °C | **This work** |

**References**

1.	R. Deivanayagam, R. Shahbazian-Yassar, “Electrochemical Methods and Protocols for Characterization of Ceramic and Polymer Electrolytes for Rechargeable Batteries” *Batteries & Supercaps* **2021**, *4*, 596–606. <https://doi.org/10.1002/batt.202000221>.

2.	I. S. Noor, S. R. Majid, A. K. Arof, “Poly(vinyl alcohol)–LiBOB complexes for lithium–air cells” *Electrochimica Acta* **2013**, *102*, 149–160. <https://doi.org/10.1016/j.electacta.2013.04.010>.

3.	P. Johansson, S. Alvi, P. Ghorbanzade, M. Karlsmo, L. Loaiza, V. Thangavel, K. Westman, F. Årén, “Ten Ways to Fool the Masses When Presenting Battery esearch” *Batteries & Supercaps* **2021**, *4*, 1785–1788. <https://doi.org/10.1002/batt.202100154>.

4.	J. Evans, C. A. Vincent, P. G. Bruce, “Electrochemical measurement of transference numbers in polymer electrolytes” *Polymer* **1987**, *28*, 2324–2328. <https://doi.org/10.1016/0032-3861(87)90394-6>.

5.	V. Landry, P. Blanchet, G. Boivin, J.-F. Bouffard, M. Vlad, “UV-LED Curing Efficiency of Wood Coatings” *Coatings* **2015**, *5*, 1019-1033. <https://doi.org/10.3390/coatings5041019>.

6.	BIOVIA Materials Studio | Dassault Systèmes, <https://www.3ds.com/products/biovia/materials-studio>.

7.	X.-D. Li, S.-Q. Feng, F. Guo, X.-Y. Liu, J.-X. Yu, Z.-W. Hou, “Predicting 1,3,5,7-tetrakis(4-aminophenyl)adamantine based covalent-organic frameworks as hydrogen storage materials” *RSC Advances* **2016**, *6*, 21517–21525. <https://doi.org/10.1039/C5RA24933C>.

8.	H. Sun, “COMPASS:  An ab Initio Force-Field Optimized for Condensed-Phase ApplicationsOverview with Details on Alkane and Benzene Compounds” *The* *Journal of Physical Chemistry B* **1998**, *102*, 7338–7364. [https://doi.org/10.1021/jp980939vh](https://doi.org/10.1021/jp980939v).

9.	I. Leontyev, A. Stuchebrukhov, “Accounting for electronic polarization in non-polarizable force fields” *Physical Chemistry Chemical Physics* **2011**, *13*, 2613–2626. <https://doi.org/10.1039/C0CP01971B>.

10.	M. Fischer, A. Heuer, D. Diddens, “Structure and Transport Properties of Poly(ethylene oxide)-Based Cross-Linked Polymer Electrolytes─A Molecular Dynamics Simulations Study” *Macromolecules* **2022**, *55*, 10229–10242. <https://doi.org/10.1021/acs.macromol.2c01795>.

11.	N. Molinari, J. P. Mailoa, B. Kozinsky, “Effect of Salt Concentration on Ion Clustering and Transport in Polymer Solid Electrolytes: A Molecular Dynamics Study of PEO–LiTFSI” *Chemistry of Materials* **2018**, *30*, 6298–6306. <https://doi.org/10.1021/acs.chemmater.8b01955>.

12.	I. V. Leontyev, A. A. Stuchebrukhov, “Electronic continuum model for molecular dynamics simulations” *The Journal of Chemical Physics* **2009**, *130*, 085102. <https://doi.org/10.1063/1.3060164>.

13.	U. Shankar, R. Gogoi, S. K. Sethi, A. Verma in *Forcefields Atomic-Scale Simulations: Materials and Applications* (Eds.: A. Verma, S. Mavinkere Rangappa, S. Ogata, S. Siengchin), Springer Nature, Singapore, **2022**, pp. 299–313. <https://doi.org/10.1007/978-981-19-3092-8_15>.

14.	F. Zhang, S. Wang, R. Fei, S. Geng, C. Huang, B. Zhao, A. Shi, Y. Jin, J. Lao, J. Yin, H. Wang, S. Liu, “Tailoring Ionic Liquid Corrosion Inhibitors for N80 Steel in Acidic Media: Anion–Cation Synergy, High-Temperature Performance, and Multiscale Mechanistic Insights” *Langmuir* **2025**, *41*, 13315–13324. <https://doi.org/10.1021/acs.langmuir.5c01038>.

15.	B. Nie, X. Zhu, P. Liu, D. Zhao, X. Liu, B. Deng, J. Lun, M. Wang, F. Qin, “Novel Insights into Gas Sorption Mechanisms in Multiscale Coal Nanopores via Molecular Simulation” *Energy Fuels* **2025**, *39*, 9373–9387. <https://doi.org/10.1021/acs.energyfuels.5c01286>.

16.	F. Chen, Z. Min, Z. Shi, P. Yang, W. Huang, “Investigation of interfacial adhesion properties and debonding behaviors between steel slag aggregates and epoxy asphalt using molecular dynamics” *Construction and Building Materials* **2025**, *482*, 141674. <https://doi.org/10.1016/j.conbuildmat.2025.141674>.

17.	W. G. Hoover, “Canonical dynamics: Equilibrium phase-space distributions” *Physical Review A* **1985**, *31*, 1695–1697. <https://doi.org/10.1103/PhysRevA.31.1695>.

18.	N. Shuichi, “Constant Temperature Molecular Dynamics Methods” *Progress of Theoretical Physics Supplements* **1991**, *103*, 1–46. <https://doi.org/10.1143/ptps.103.1>.

19.	R. W. Hockney, J. W. Eastwood, *Computer Simulation Using Particles*, CRC Press, Boca Raton, **2021**. <https://doi.org/10.1201/9780367806934>.

20.	M. L. Connolly, “Analytical molecular surface calculation” *Journal of Applied Crystallography* **1983**, *16*, 548–558. <https://doi.org/10.1107/S0021889883010985>.

21.	B. Delley, “From molecules to solids with the DMol3 approach” *The Journal of Chemical Physics* **2000**, *113*, 7756–7764. <https://doi.org/10.1063/1.1316015>.

22.	B. Delley, “An all-electron numerical method for solving the local density functional for polyatomic molecules” *The Journal of Chemical Physics* **1990**, *92*, 508–517. <https://doi.org/10.1063/1.458452>.

23.	A. M. DiCiccio, G. W. Coates, “Ring-Opening Copolymerization of Maleic Anhydride with Epoxides: A Chain-Growth Approach to Unsaturated Polyesters“ *Journal of American Chemical Society* **2011**, *133*, 10724–10727. <https://doi.org/10.1021/ja203520p>.

24.	J. M. Hutchinson, “Determination of the glass transition temperature” *Journal of Thermal Analysis and Calorimetry* **2009**, *98*, 579–589. [https://doi.org/10.1007/s10973-009-0268-0](https://doi.org/10.1007/s10973-009-0268-0%20).

25.	M. G. Nam, J. Moon, M. Kim, J. K. Koo, J.-W. Ho, G. H. Choi, H. J. Kim, C.-S. Shin, S. J. Kwon, Y.-J. Kim, H. Chang, Y. Kim, P. J. Yoo, “p-Phenylenediamine-Bridged Binder-Electrolyte-Unified Supramolecules for Versatile Lithium Secondary Batteries” *Advanced Materials* **2024**, *36*, 2304803. <https://doi.org/10.1002/adma.202304803>.

26.	E. O. Stejskal, J. E. Tanner, "Spin Diffusion Measurements: Spin Echoes in the Presence of a Time Dependent Field Gradient" The journal of chemical physics 1965, 42, 288-292. <http://dx.doi.org/10.1063/1.1695690>.

27.	X. Xie, P. Zhang, X. Li, Z. Wang, X. Qin, M. Shao, L. Zhang, W. Zhou, “Rational Design of F-Modified Polyester Electrolytes for Sustainable All-Solid-State Lithium Metal Batteries” *Journal of the American Chemical Society* **2024**, *146*, 5940–5951. <https://doi.org/10.1021/jacs.3c12094>.

28.	X. Xie, Z. Wang, S. He, K. Chen, Q. Huang, P. Zhang, S.-M. Hao, J. Wang, W. Zhou, “Influencing Factors on Li-ion Conductivity and Interfacial Stability of Solid Polymer Electrolytes, Exampled by Polycarbonates, Polyoxalates and Polymalonates” *Angewandte Chemie International Edition* **2023**, *62*, e202218229. <https://doi.org/10.1002/anie.202218229>.

29.	Y. Seo, Y.-C. Jung, M.-S. Park, D.-W. Kim, “Solid polymer electrolyte supported by porous polymer membrane for all-solid-state lithium batteries” *Journal of Membrane Science* **2020**, *603*, 117995. <https://doi.org/10.1016/j.memsci.2020.117995>.

30.	W. Zhou, Z. Wang, Y. Pu, Y. Li, S. Xin, X. Li, J. Chen, J. B. Goodenough, “Double-Layer Polymer Electrolyte for High-Voltage All-Solid-State Rechargeable Batteries” *Advanced Materials* **2019**, *31*, 1805574.  <https://doi.org/10.1002/adma.201805574>.
